# SophosQM: Accurate Binding Affinity Prediction in Compound Optimization

**DOI:** 10.1021/acsomega.2c08132

**Published:** 2023-04-20

**Authors:** Riccardo Guareschi, Iva Lukac, Ian H. Gilbert, Fabio Zuccotto

**Affiliations:** Drug Discovery Unit, Wellcome Centre for Anti-Infectives Research, Division of Biological Chemistry and Drug Discovery, University of Dundee, Dow Street, Dundee DD1 5EH, United Kingdom

## Abstract

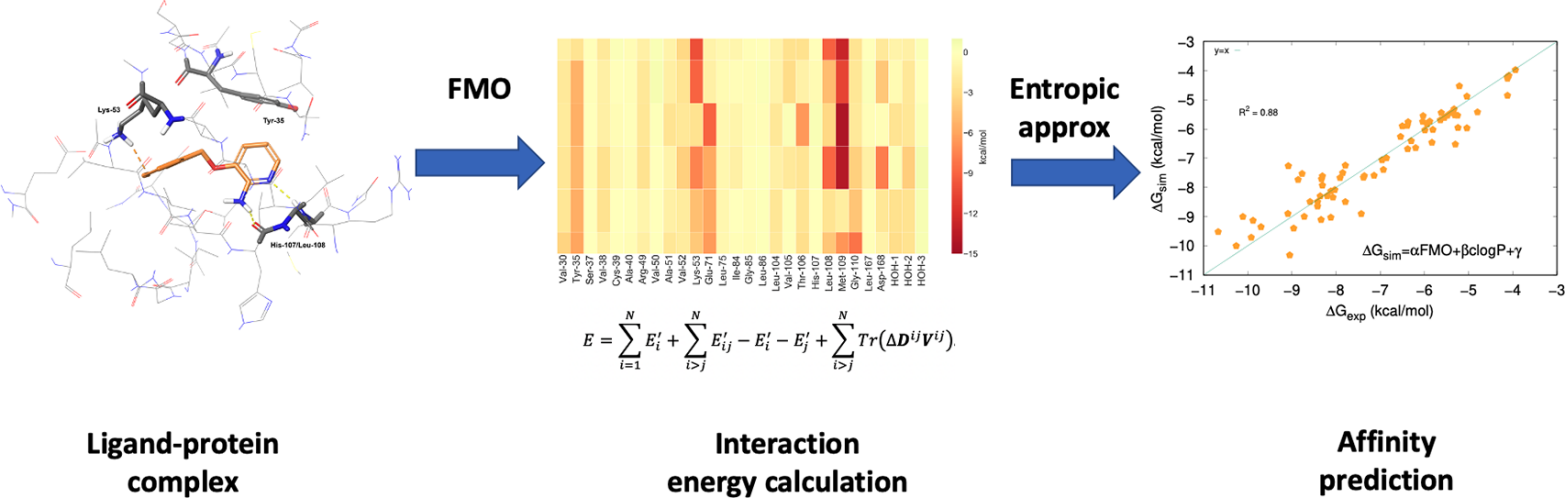

The optimization of compounds’ binding affinity for a biological target is a crucial aspect of the drug development process. Being able to accurately predict binding energies in advance of synthesizing compounds would have a massive impact on the speed of the drug discovery process. The ideal binding affinity prediction method should combine accuracy, reliability, and speed. In this paper, we present SophosQM, a quantum mechanics (QM)-based approach, which can accurately predict the binding affinities of compounds to proteins. The binding affinity predictive models generated by SophosQM are based on the fragment molecular orbital (FMO) method to estimate the enthalpic component of the binding free energy, and a macroscopic descriptor, clog *P*, is used as an approximation of the entropic component. The affinity prediction is performed using multilinear regression, fitting the experimental values against the FMO-computed enthalpic term and clog *P*. The quality of the prediction can be assessed in terms of the correlation coefficient between experimental and predicted values. In this work, the method’s reliability and accuracy are exemplified by applying SophosQM to 70 compounds binding to six different targets of pharmaceutical relevance. Overall, the results show a very satisfactory performance with a global correlation coefficient in the order of 0.9. Our predictions also show a satisfactory performance compared to data based on free energy perturbation. Finally, SophosQM can also be applied in high-throughput mode by using semiempirical QM methods to evaluate large portions of chemical space, while retaining a good level of accuracy, but decreasing the computing time to just a few seconds per compound.

## Introduction

The interaction between a small molecule and its biological target is at the center of the drug discovery paradigm. Safe and efficacious drugs are the result of a lengthy and resource-intensive multiparameter optimization process, where structural changes are introduced into an initial hit compound to optimize its binding affinity and achieve a favorable physicochemical/ADME property profile. Normally this is achieved following multiple iterations of the design, make, test, analyze (DMTA) cycle. In the context of target-based, structurally enabled projects, the availability of high-resolution structural data provides a unique opportunity to study the molecular recognition process at an atomic level. Understanding ligand–protein binding interactions is critical to rationalizing potency and selectivity and ultimately guide the molecular design of new compounds and improve the efficiency of the DMTA cycle.

Over the last few decades, the in silico analysis of the binding interactions and the prediction of interaction energies relied heavily on a combination of visual inspection and force-field-based molecular mechanics methods, such as docking and molecular dynamics. Force fields are however poorly equipped to capture the complexity and diversity of the molecular interactions^1^ with a number of nonclassical interactions not being adequately parameterized^2^ (e.g., CH−π, halogen−π, cation−π interactions, and nonclassical hydrogen bonds).

Quantum mechanics (QM), within the obvious approximations inherent to the choice of the basis set, and the flavor of the approach chosen, offers the most accurate methods to analyze molecular interactions. Unfortunately, high accuracy comes usually at the cost of very long computational times and unfavorable scaling with respect to the size of the system studied. Since the binding pocket of a biological target includes hundreds of atoms, traditional QM methods could not match timelines and the scale required to impact the typical drug discovery project.

However, the significant advances experienced in computing in the last decades and new methods recently developed have greatly expanded the applicability of QM approaches in drug discovery. In particular, the *ab initio* fragment molecular orbital (FMO) method^3−6^ offers substantial resource saving over traditional QM methods. FMO is a “divide and conquer” approach, where computing efficiency is obtained by dividing large biological systems into smaller fragments and then performing the QM calculation on each fragment–ligand pair. The resulting pairwise interaction energy (PIE) between each of the fragments and the ligand can be combined to derive the total interaction energy of the ligands with the target.

The decomposition analysis of the PIE (PIE decomposition analysis—PIEDA)^7^ allows the derivation of four different interaction energy terms: electrostatic, charge transfer, dispersion, and exchange-repulsion. These provide a deeper residue-by-residue insight into the nature of the ligand interaction with the target. The electrostatic and charge transfer terms are dominant in interaction energies for H-bonds, polar (favorable and unfavorable) interactions, and salt bridges. The dispersion term is more prominent in hydrophobic and van der Waals interactions. The exchange-repulsion term describes the steric repulsion between electrons of different atoms accounting for steric clashes.

The FMO method provides a very powerful tool to rationalize potency and explain structure–activity relationships (SAR),^8−10^ and the total pair interaction energy (tPIE) obtained has been used as a scoring function to assess newly designed compounds. It displays a significant correlation with the experimentally derived ligand binding energy as it describes the enthalpic component. However, it does not consider the entropic and solvation contribution to the interaction energy.

Recently, in their theoceptor model, Lukac et al.^11^ have shown that all of the nonenthalpic contributions to ligand–protein binding can be captured by the logarithm of the ligand’s partition coefficient (log *P*), a molecular parameter that can be easily measured or accurately predicted. According to this approach, it is assumed that molecules that establish strong electrostatic interactions with their target have a binding free energy dominated by the enthalpic term. The magnitude of such interactions can range from ∼5 kcal/mol for hydrogen bonds to hundreds of kcal/mol for ionic pairs. Cumulatively, the enthalpy gain obtained through such contacts offsets the desolvation and entropy loss penalties. Overall, this gives rise to a favorable binding free energy. On the other hand, large lipophilic compounds will have a favorable desolvation entropy and small enthalpic penalties. As a result, the binding of this class of compounds is driven by favorable changes of entropy and close-range van der Waals contacts.^12^ This led to the approximation of all of the nonenthalpic terms with the macroscopic descriptor log *P*.

Therefore, the binding affinity of the ligand can be expressed as a multilinear regression based on interaction energy and log *P* (eq 1) where the coefficients α, β, and γ are constant for the ligand–protein complex and can be derived by fitting a set of experimental affinity values.
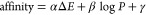
1

Using Δ*E* values derived by QM calculations as part of their theoceptor approach, the authors obtained significant correlations between calculated and experimental values for compounds binding to the enzyme Lactate Dehydrogenase A (LDHA). Due to computing limitations, conventional quantum mechanics is severely restricted in the number of atoms from the ligand and residues from the protein that can be realistically analyzed.

With the aim of delivering the accuracy of QM methods, within the pressing time frame of drug discovery projects, we developed SophosQM. This combines the more rapidly calculated FMO-derived interaction energy (tPIE), with a theoceptor-like approach. With FMO we can easily expand the number of atoms to include all of the residues defining the binding site and reduce the calculation time. By accurately predicting binding affinity, SophosQM is an effective tool to prioritize target compounds for synthesis and assist critical decisions within drug development projects.

In this work, we present a study to assess the performance of SophosQM in evaluating the binding affinity of a selection of fragment-like compounds. Due to their low affinity and low molecular complexity, fragments are a particularly challenging test case as the in silico methods need to be sensitive to small molecular variations and able to differentiate between low-affinity compounds. For this purpose, we focused on a set of data, previously used to benchmark free energy perturbation methods, which are currently considered to be the gold standard by the pharmaceutical industry for the prediction of compounds’ potency. Our work shows that SophosQM performs at the level of accuracy and speed required to support compounds optimization projects in drug discovery and can offer a valid alternative to free energy perturbation (FEP) methods.

During the compound optimization process, the chemical space surrounding the initial hit can be very large. Modern synthetic chemistry has made available an unprecedented repertoire of chemical reactions that allow the introductions of a very diverse set of functional groups on different positions of the initial hit scaffold. The systematic enumeration of all of the possible compounds that can be derived by medicinal chemistry transformation can lead to 10^4^–10^6^ potential target compounds. The size of this close analogous chemical space makes it really challenging for medicinal chemists to identify the groups that optimally match the steric and pharmacophoric requirement of the biological target. For this reason, it is very important to be able to deploy accurate affinity prediction methods on a large scale. To address this problem, SophosQM can be deployed in high-throughput mode where FMO relies on semiempirical quantum mechanics (SQM) calculations instead of traditional QM to derive the receptor–ligand interaction energy in a matter of seconds. For the purpose of this work, we discuss only the results obtained using FMO-QM calculations. However, Morao et al.^13,14^ have shown that FMO-SQM results are consistent with those obtained with FMO-QM, but the calculation time is reduced by thousands of times (from hours to seconds per compound).

## Experimental Section

### FMO Theoretical Basis

SophosQM is used to evaluate the interaction energy of the compound with the target. The FMO method has been proven to calculate efficiently the energy of large-sized molecular systems and it is particularly appealing to evaluate the energy of ligand–receptor interactions.^3,6,9,15^ In this application, a protein–ligand complex is fragmented mapping one fragment to the ligand and one to each of the residues of the target binding site.

Following the derivation of the FMO equations^3−5,16^ the total energy, *E*, of a system of *N* fragments is given by
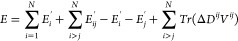
2

The algorithm of the FMO method in the so-called 2-body expansion requires a first set of QM calculations where the energy and electron density of each fragment (monomer) is derived. Then, a second set of QM calculations is performed to obtain the energy and electron densities of every possible combination of two fragments (dimer). Since the orbitals of the monomers are perturbed during the calculations on the dimers, the approach introduces polarization effects on each fragment which result from the presence of all of the other fragments of the system. In our case, all of the dimers and monomers are described by single-determinant wavefunctions with closed-shell electronic structure. In eq 2, *E*_i_^′^ and *E*_*ij*_^′^ are the energies of single fragments and pair fragments from which the interaction with the external potential has been subtracted, i.e., *E*_*i*_^′^ = *E*_*i*_ – Tr(***D**^i^**V**^i^*), ***D**^i^* being the density matrix and ***V**^i^* being the embedding electrostatic potential of the monomer *i*; Δ***D**^ij^* = ***D**^ij^* – ***D**^i^* – ***D**^j^* is the difference between the density matrix of a dimer and the density matrices of the monomers. For a single-determinant wavefunction with a closed-shell configuration with *K* molecular orbitals, which is expressed as a linear combination of a basis set of atomic orbitals (LCAO), the density matrix is defined as , where *C* are the expansion coefficients of the LCAO. The interaction energy between two fragments is given by eq 3
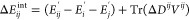
3

Following this equation, the energy of a fragment can be written as the sum of all of the interactions established with each one of the other fragments present (eq 4). In particular, we are interested in the case when the index *i* corresponds to the ligand so that its interaction energy with the remaining of the system will be given by
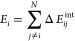
4

A set of different ligands surrounded by the same set of amino acids can be ranked by directly comparing the different interaction energies obtained from eq 4. This term does not account for the variations in the internal energy of the ligand between its free and bound state (i.e., the geometrical strain of the ligand is not considered) and does not account for the electron redistribution in the complex upon ligand binding. This means that E_i_ does not correspond to the binding enthalpy of the ligand, Δ*H*. Nonetheless, it is observed that a proportionality subsists between the sum of the pair interactions and Δ*H*.^17,18^ This lets us introduce the following approximation for the variation of binding enthalpy for a certain ligand, *i*
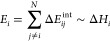
5

Within the FMO formalism, it is also possible to divide the Δ*E_ij_^nt^* in different components corresponding to different kinds of physical interactions. In the pair interaction energy decomposition analysis (PIEDA), the Δ*E_ij_^int^* is written as the sum of electrostatic (ES), exchange-repulsion (EX), charge transfer (CT), and dispersion interactions (Disp), namely
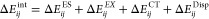
6

This decomposition is particularly appealing in order to understand the nature of the protein–ligand binding and to easily identify the effect of the introduction of a certain substituent on a ligand scaffold within a congeneric series.

Clearly, in order to simulate a binding free energy, an approximation for the entropic term must be also introduced. In the present work, we aim at replacing the calculation of the complex *T*Δ*S* quantity with a molecular descriptor that can be easily obtained, and it is able to incorporate the entropic effects. Our starting choice is to use the lipophilicity of the ligand for this purpose. The lipophilicity is expressed as the logarithm of the calculated octanol/water partition coefficient, clog *P*. The parameter clog *P* can be quickly estimated on the basis of the 2D structure of a ligand using different software. This choice proved to be very efficient in similar applications^11^ and represents our reference parameter to incorporate entropic effects in our estimations of Δ*G*.

Given a set of ligands for which the experimental binding affinity Δ*G*_exp_ is known, we propose to obtain a simulated value Δ*G*_sim_ by linearly fitting Δ*G*_exp_ with the *E*_*i*_ values obtained by FMO calculations and the clog *P*_i_ terms according to this equation:
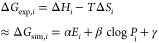
7

with α, β, and γ being the fitting parameters obtained by linear regression. Once the fitting parameters have been obtained, they can be also used to predict the Δ*G* of ligands for which the experimental value is not known, under the assumption that such ligands share the same binding mode and belong to similar chemical series as those used to build the linear regression.

The quality of this approximation is assessed in terms of the adjusted correlation coefficient, *R*^2^, between the Δ*G*_sim_ and the Δ*G*_exp_ values. For a multiparameter linear fit, the adjusted *R*^2^ reflects the quality of a fit independently of the number of linear parameters used and therefore is the best metric to assess the results obtained with this protocol. Another performance metric is the absolute error between the simulated and experimental values averaged over the number of compounds available for the fitting, *N*_mol_
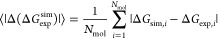
8

### Computational Details

For each system, at least one reference X-ray structure with one co-crystallized ligand is available in the PDB database. The protein–ligand structures were initially processed using the Protein Preparation module in the Schrödinger suite using the default parameters. During this step, the water molecules within 5 Å of the co-crystallized ligand are retained as part of the structure and their hydrogen bond network is optimized. The complexes are generated by molecular docking with a grid built around the co-crystallized ligand. The docking is performed with GLIDE from the Schrödinger suite of programs using the SP (standard precision) algorithm.^19−21^ The poses selected are those that most closely align the core of the ligand with the core of the ligand present in the experimental structure. If multiple possible poses can be generated by rotations around σ-bonds, all of the geometries are used for the subsequent FMO calculations and only the one yielding the lowest value of FMO energy according to eq 4 is used in the linear fit.

The complexes thus generated are minimized with the OPLS3e force field^22^ using MacroModel from the Schrödinger suite of programs.^23^ The minimization is performed with a restraint of 100 kJ/mol on the ligand atoms to ensure that the position of the core remains closely aligned on the core of the crystallographic ligand. The RMSD between the pose generated by docking and the pose observed in the crystal structure was used to assess the accuracy of the docking process for every native ligand (Table S7). From the minimized complexes, the atoms of the residues within approximately 5 Å from the ligand are selected for the FMO calculations. Acetyl (ACE) and methyl-amine (NME) capping groups are introduced to saturate the valence of the atoms at the boundary of the FMO selection.

The definition of the fragments for the FMO calculations is performed using the program FACIO.^24,25^ One fragment corresponds to one amino acid shifted by one C_α_, i.e., the fragmentation for a chain of connected amino acids always truncates the C_α_–C bond. The ligand and the water molecules, when present, are assigned to a single fragment each.

The FMO calculations are performed using GAMESS.^26,27^ As a default we use the two-body implementation of the FMO method with the second-order Møller–Plesset perturbation theory (MP2) as the quantum mechanical level of theory.^28^ The basis set employed is 6-31G*.^29^ This choice is generally regarded as an efficient way to combine accuracy and computational cost within the framework of the FMO method.^15^ In the high-throughput mode, which uses semiempirical quantum mechanics (SQM) approaches, we use the third-order electron density expansion DFTB3 with 3ob parameters (data not shown).^30^ DFTB3 is a recent extension of the self-consistent-charge density functional tight-binding method (SCC–DFTB) and derived from a third-order expansion of the density functional theory (DFT) total energy around a given reference density. The clog *P* values are calculated using Volsurf.^31^

The experimental binding energies used in this study, are taken from Steinbrecher et al.^32^ In this work, focused on assessing FEP, different measures of affinity have been collected from several experimental publications and converted to Δ*G*. We choose to keep this set of values as reference experimental affinity values to have a global comparison across all of the systems studied using Δ*G*_exp_^sim^ in addition to the adjusted *R*^2^. To enable comparison, we have not changed the names of the ligands from the original publication.^32^ The details of the residues included in the FMO calculations and the size of each system are reported in the Supporting Information.

Our study is based on a selection of 70 compounds binding, to six different targets of pharmaceutical interest (Table 1). For every target, the binding affinity of close analogues belonging to the same chemical series was evaluated. The targets are the bacterial DNA ligase (DNA ligase), Myeloid Cell Leukemia 1 (MCL-1), Major urinary protein I (MUP-1), Janus Kinase 2 (JAK-2), Heat shock protein 90 (HSP90), and p38α MAP kinase (p38). This choice follows the selection of ligand–protein interactions analyzed in ref (32). We decided to exclude the Lysozyme, due to the high flexibility within the binding pocket, which makes it less suitable for a fixed-geometry approach such as ours, and human lactate dehydrogenase isoform A (LDH), since no quantitative binding energy was reported for the binders of this enzyme, but only a percentage of inhibition. All chemical series considered had a neutral charge, with the exception of the chemical series binding to MCL-1 which is characterized by a formal charge of −1.

**Table 1:** Structural Data for the Systems Considered in This Work

	system	species	PDB ID	resolution (Å)
A	DNA ligase	*Staphylococcus aureus*	4CC5	1.9
B	MCL-1	*Homo sapiens*	4HW3	2.4
C	MUP-1	*Homo sapiens*	1I06	1.9
D	JAK-2	*Homo sapiens*	3E62/3E64	1.9/1.9
E	HSP90	*Homo sapiens*	3FT8	2.0
F	p38	*Homo sapiens*	1W7H	2.2

For each system studied, we closely followed the setup described in ref (32) in order to obtain a reliable geometry to start our FMO calculations. Since the FMO method is very sensitive to the atomic coordinates chosen, it is important to work with geometries that are well minimized and as close as possible to the crystallographic reference available. Table 1 summarizes the structural data considered for the systems studied in this work.

For each system under investigation, in-house-developed scripts were used to generate the input files for FACIO and GAMESS. Similarly, in-house scripts were used to analyze FMO GAMESS output and generate ligand–protein interaction heat map displaying the interaction energies of each compound and the single residues in the binding sites.

## Results

### SophosQM Predictive Models

#### DNA Ligase

This series contains a set of 11 ligands that explore scaffold modifications of compound DNA_lig03, co-crystallized in PDB ID 4CC5 (Figure S1). The substitutions involve the chlorine atom with other lipophilic groups as well as the replacement of the triazole ring with a 6-N indazole for DNA_lig11, DNA_lig12, and DNA_lig13.

**Figure 1:**
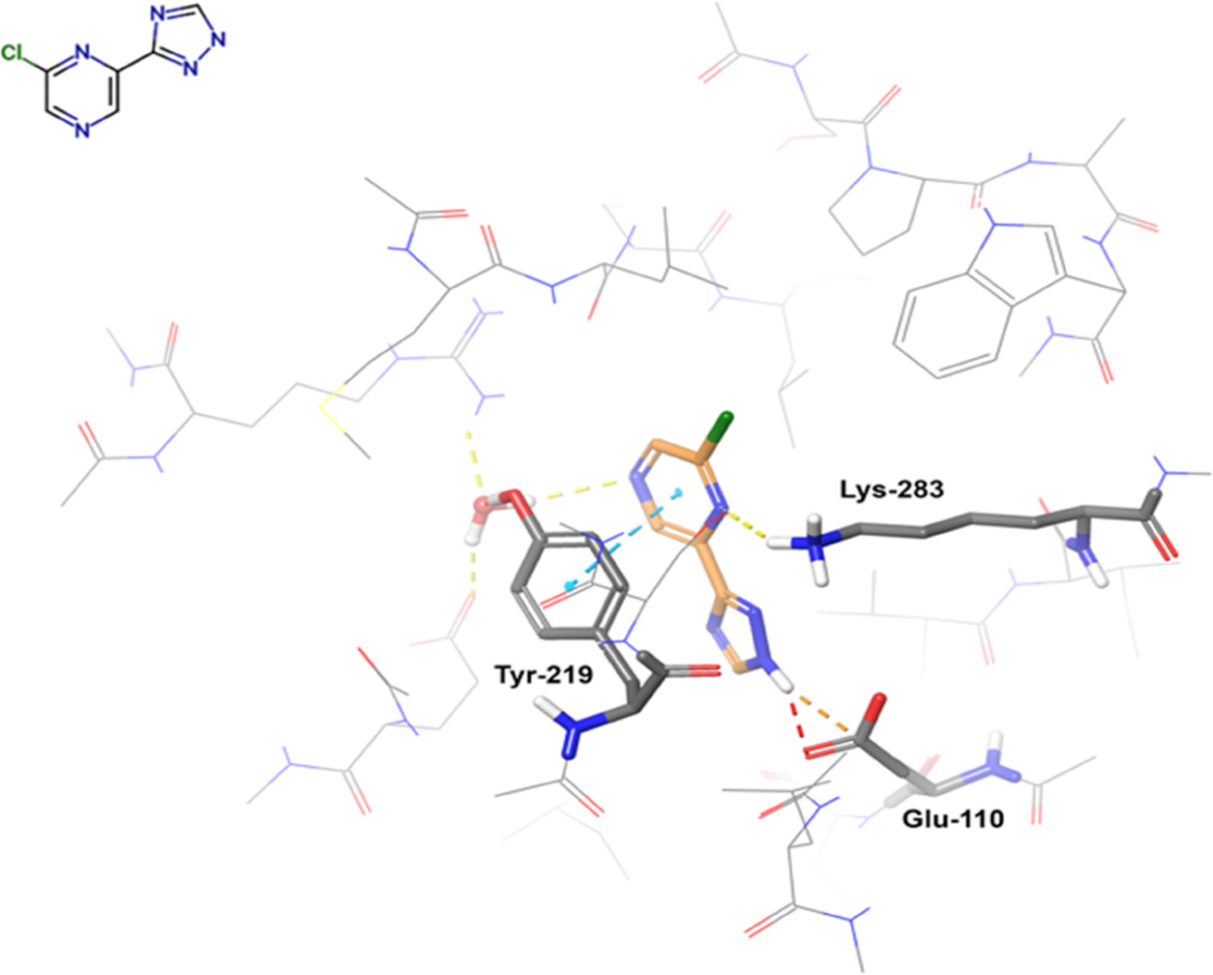
Binding pose of DNA_lig03 in DNA ligase (PDB ID 4CC5). The ligand is represented by orange sticks and the protein by gray thin lines. The protein residues establishing direct contacts with the ligand are shown in gray sticks. Nonpolar hydrogens are not shown.

The binding pose of DNA_lig03, as experimentally determined, is stabilized by a combination of hydrophobic and polar contacts with the binding site residues (Figure 1). In particular, we noticed a π–π stacking with Tyr-219, a dipole–charge interaction between Lys-283 and the pyrazine ring, and a dipole–charge interaction between the triazole ring and Glu-110. This geometry justifies our protonation choice that assigns one H atom to the nitrogen of the triazole ring pointing toward this anionic side chain. The binding site of the reference X-ray structure is solvated by 17 water molecules that create an extended H-bond network. The arrangement of the water molecules is suitable to accommodate all of the 11 ligands studied without steric clashes. For this reason, all of the waters are retained in the FMO calculations, even if only one of them is directly involved in a H-bond contact with the ligand. The choice of retaining a large ensemble of waters is based on the assumption that a more realistic description of the embedding potential can be achieved in this way. This is particularly relevant if we consider that the FMO calculations do not include implicit forms of solvation. This choice reflects our intent to minimally disrupt the experimental structure and to avoid the introduction of additional solvation terms which could be unrealistic (for instance in the case of residues placed inside lipophilic pockets).

**Figure 2:**
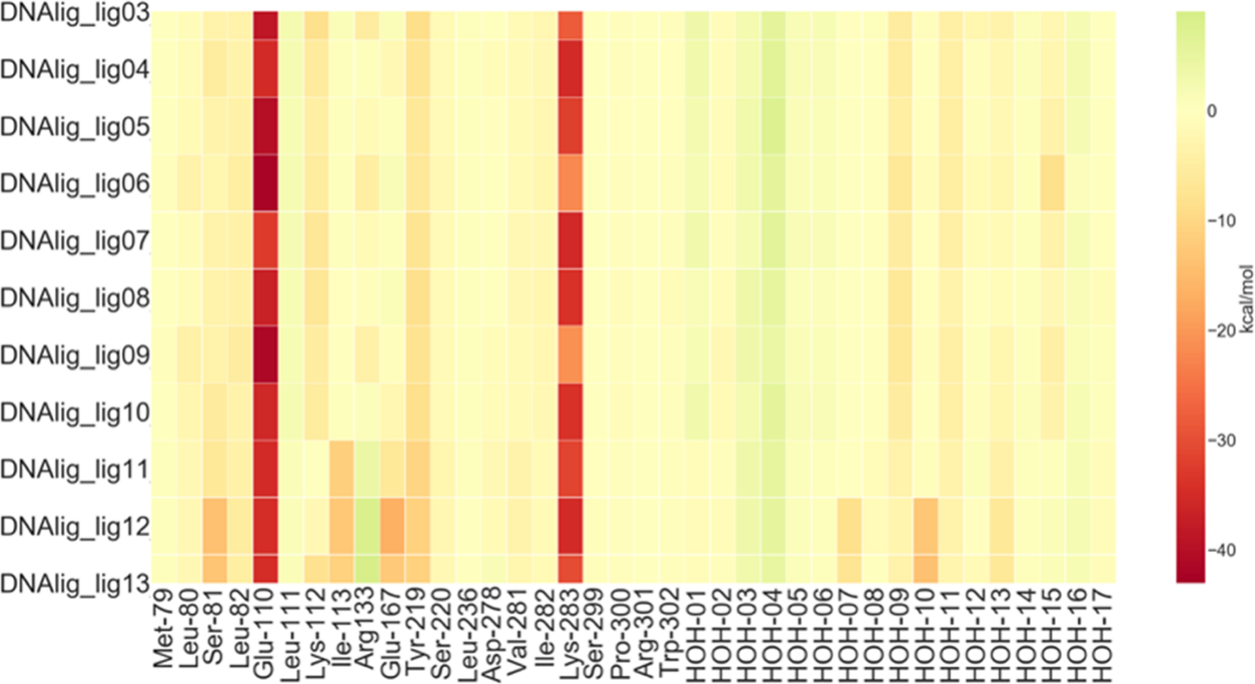
Ligand–protein interaction energy heat map of DNA ligase. The values plotted (kcal/mol) are obtained from eq 3, where one of the two indexes corresponds to the ligand and the other index to the fragments of the system.

The ligand–protein interaction heat map obtained from the FMO calculations (Figure 2) shows an overall conserved pattern. The electrostatic contacts with Glu-110 and Lys-283 are, as expected, the most stabilizing ones. For DNA_lig11, DNA_lig12, and DNA_lig13 an additional H-bond is established with the backbone of Ile-113 and a mild repulsion with Arg-133 arises. The increment of the molecular size in these three compounds is directly linked to a lower value of experimental binding affinity, which also appears in the FMO interaction energies.

Overall, we observe a good correlation between the Δ*G*_exp_ and the FMO interaction energy, which suggests that the enthalpic contributions play a substantial role in the ligand binding (Figure S2-A). On the other hand, there is absolutely no correlation between the experimental binding affinity and the simulated lipophilic term, clog *P* (Figure S2-B). For this reason, we conclude that the binding is exclusively dominated by the enthalpic contributions.

**Figure 3:**
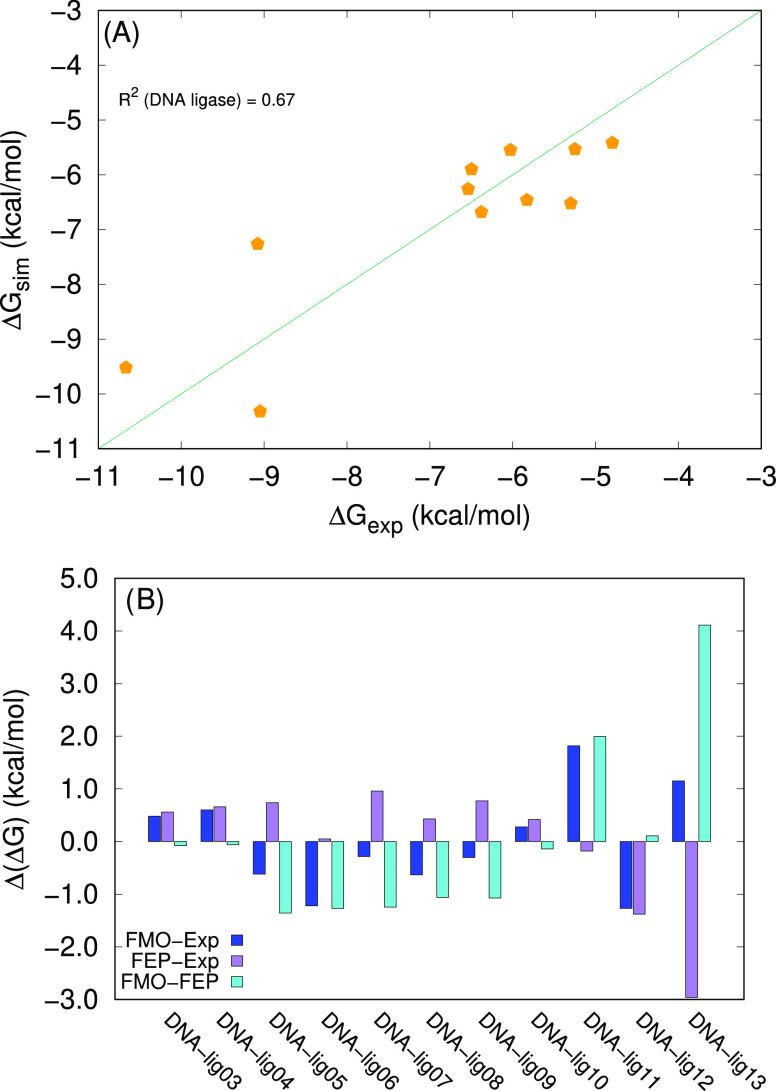
(A) Correlation between FMO-based simulated values and experimental Δ*G* values for DNA ligase. The diagonal line corresponds to Δ*G*_exp_ = Δ*G*_sim_. (B) Differences between the FMO-based predictions (our data), the FEP-based predictions, and Δ*G*_exp_ for DNA ligase. The experimental values and the FEP-based predictions are taken from ref (32).

The resulting predictions of Δ*G*_sim_ are in acceptable agreement with the experimental values (Figure 3A) with an average absolute error in the order of about 0.8 kcal/mol (Figure 3B). With our predicted data, it is still possible to identify two classes of compounds: The three at the lowest affinity (approximatively −11 < Δ*G*_sim_ < −9 kcal/mol) and all of the other with Δ*G*_sim_ > −7 kcal/mol. Our predictions show a variable agreement with the data simulated by FEP.^32^ The average difference with respect to the FEP values is in the order of 1.1 kcal/mol. Interestingly, we estimate a Δ*G*_sim_ for DNA_lig13 which is much closer to the experimental value than the FEP prediction, where the error is in the order of 3 kcal/mol.

#### MUP-I

This series contains a set of seven ligands that explore scaffold modifications of the small-molecule co-crystallized in PDB ID 1T06 (Figure S3). This structure corresponds to the complex between MUP-I and ligand MUP_lig01. The substitutions involve the aliphatic branches attached at position 2 on the 4,5-dihydrothiazole ring.

**Figure 4:**
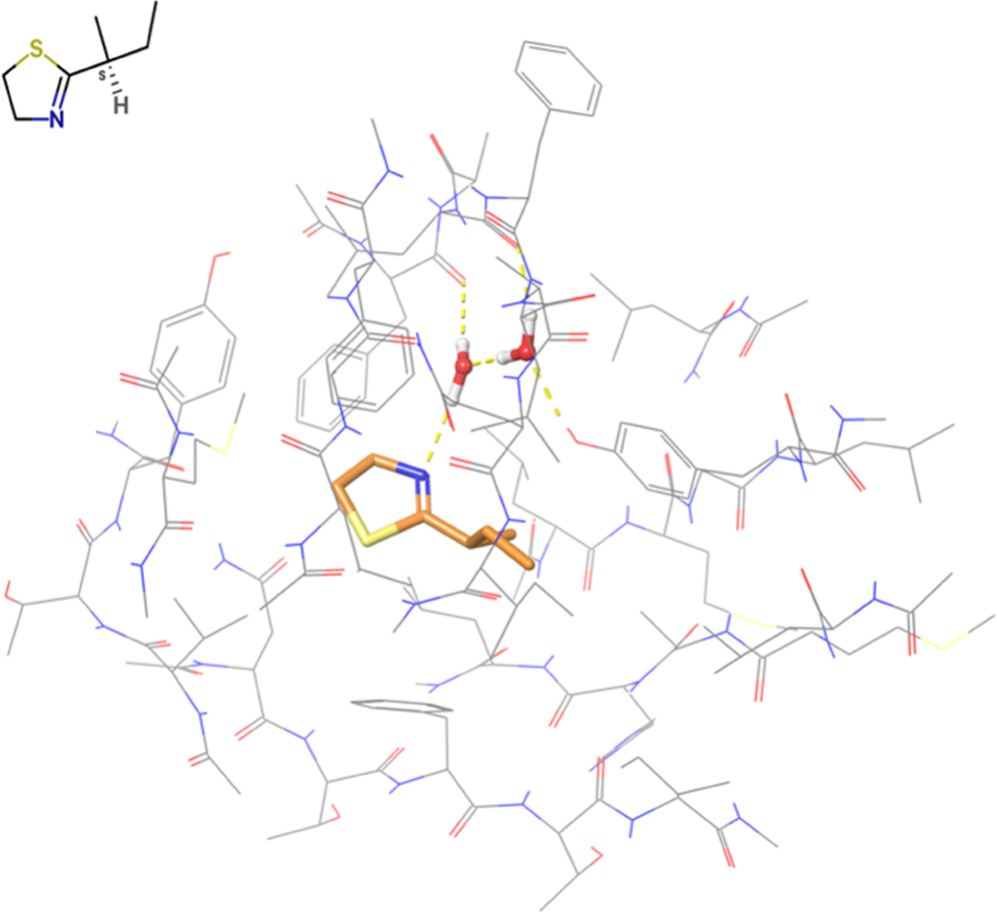
Binding pose of MUP_lig01 in MUP-I (PDB ID 1T06). The ligand is represented by orange sticks and the protein by thin gray lines. Nonpolar hydrogens are not shown.

The experimental binding pose reveals that the small ligand is stabilized mostly by hydrophobic contacts. In addition, one water molecule establishes a H-bond between the N atom of the 4,5-dihydrothiazole ring and the backbone of Phe-56. Another water molecule, completes a small Hydrogen bond network, placing itself between the side chain of Tyr-138, the backbone of Leu-58, and the water molecule previously described. The two water molecules are included in the FMO calculations. The binding pose of MUP_lig01 in MUP-I is shown in Figure 4.

**Figure 5:**
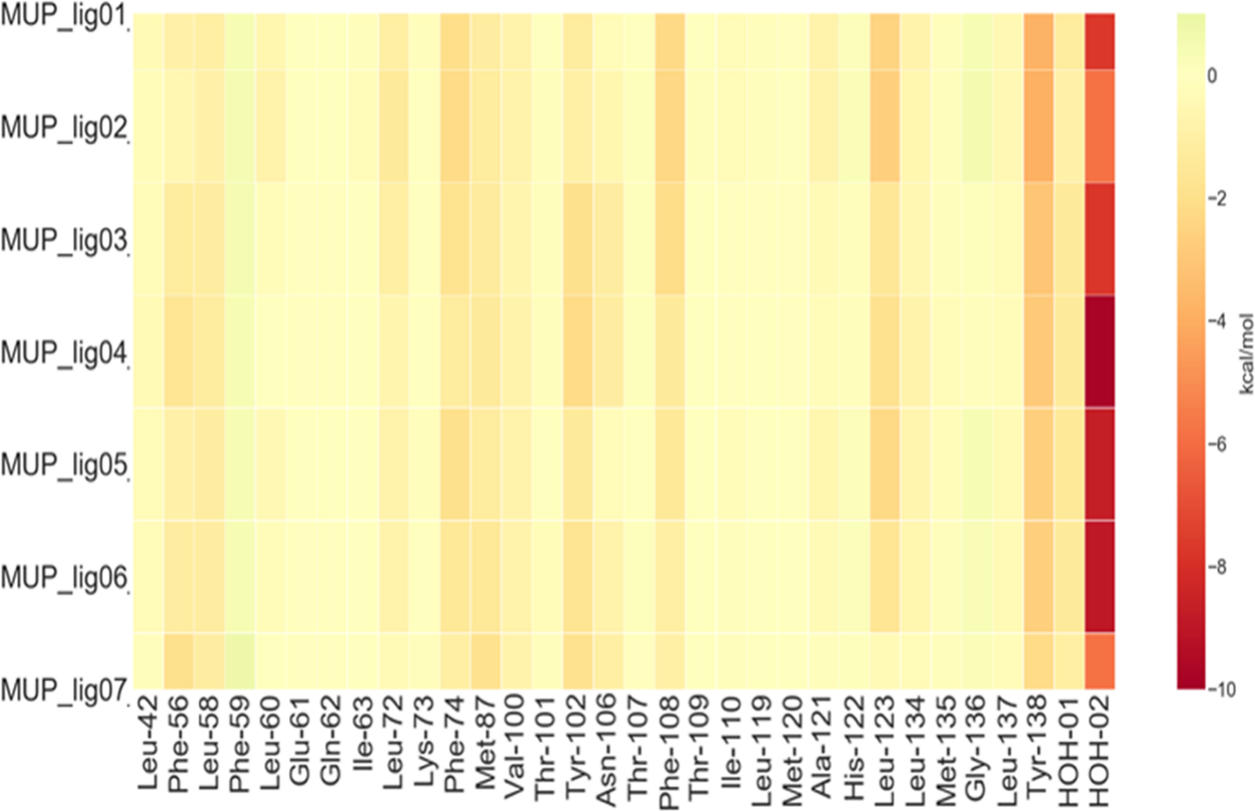
Ligand–protein interaction energy heat map of MUP-I. The values plotted (kcal/mol) are obtained from eq 3, where one of the two indexes corresponds to the ligand and the other index to the fragments of the system.

As expected, the protein–ligand interaction heatmap produced by the FMO calculations shows small differences across the ligands considered (Figure 5). The differences come essentially from the aliphatic branching of the substituents.

The low molecular weight of these fragments is associated with a lower value of the FMO energy, which reasonably correlates with the experimental affinity (Figure S4-A). This correlation comes essentially from the additive contributions of bigger substituents that establish more lipophilic contacts with the binding pocket. Looking at Figure S4-B, it appears clear that the driving force of the binding is indeed the lipophilicity of the compounds studied, rather than the enthalpic term.

**Figure 6:**
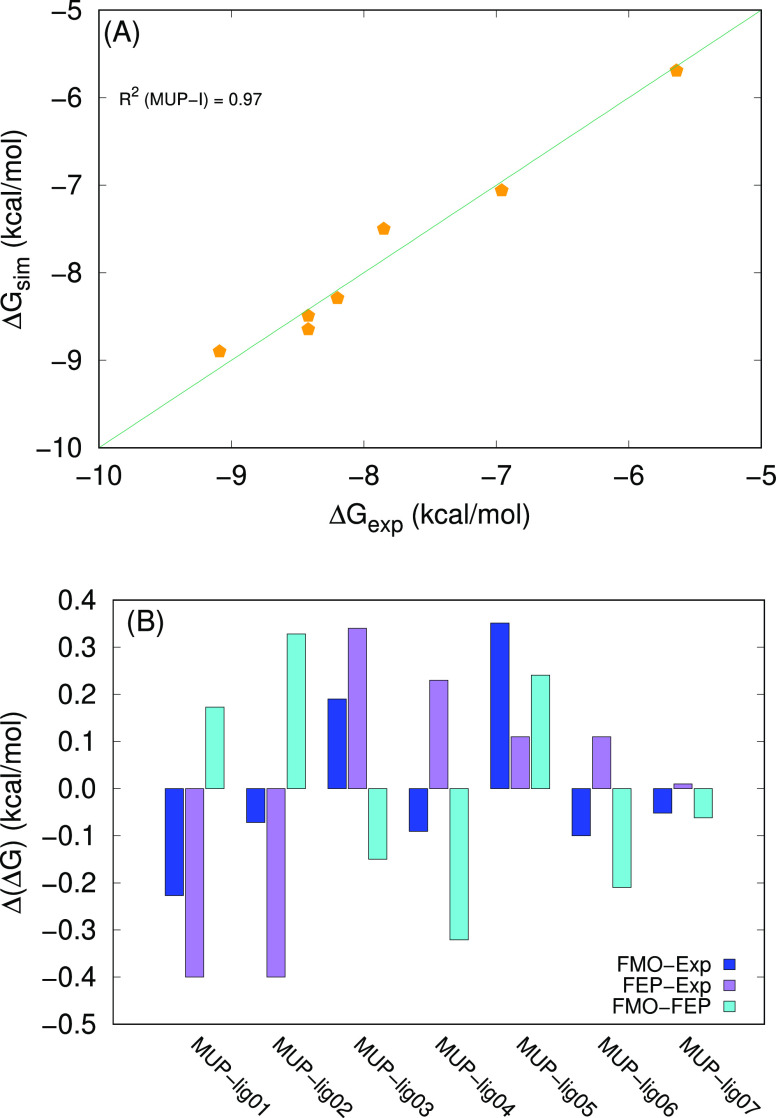
(A) Correlation between FMO-based simulated values and experimental Δ*G* values for MUP-I. The diagonal line corresponds to Δ*G*_exp_ = Δ*G*_sim_. (B) Differences between the FMO-based predictions (our data), the FEP-based predictions, and Δ*G*_exp_ for MUP-I. The experimental values and the FEP-based predictions are taken from ref (32). Notice that the errors are smaller than 0.4 kcal/mol in absolute value.

The resulting predictions of Δ*G*_sim_ are in excellent agreement with the experimental values (Figure 6A) and with the predictions generated by FEP^32^ (Figure 6B)

#### HSP90

**Figure 7:**
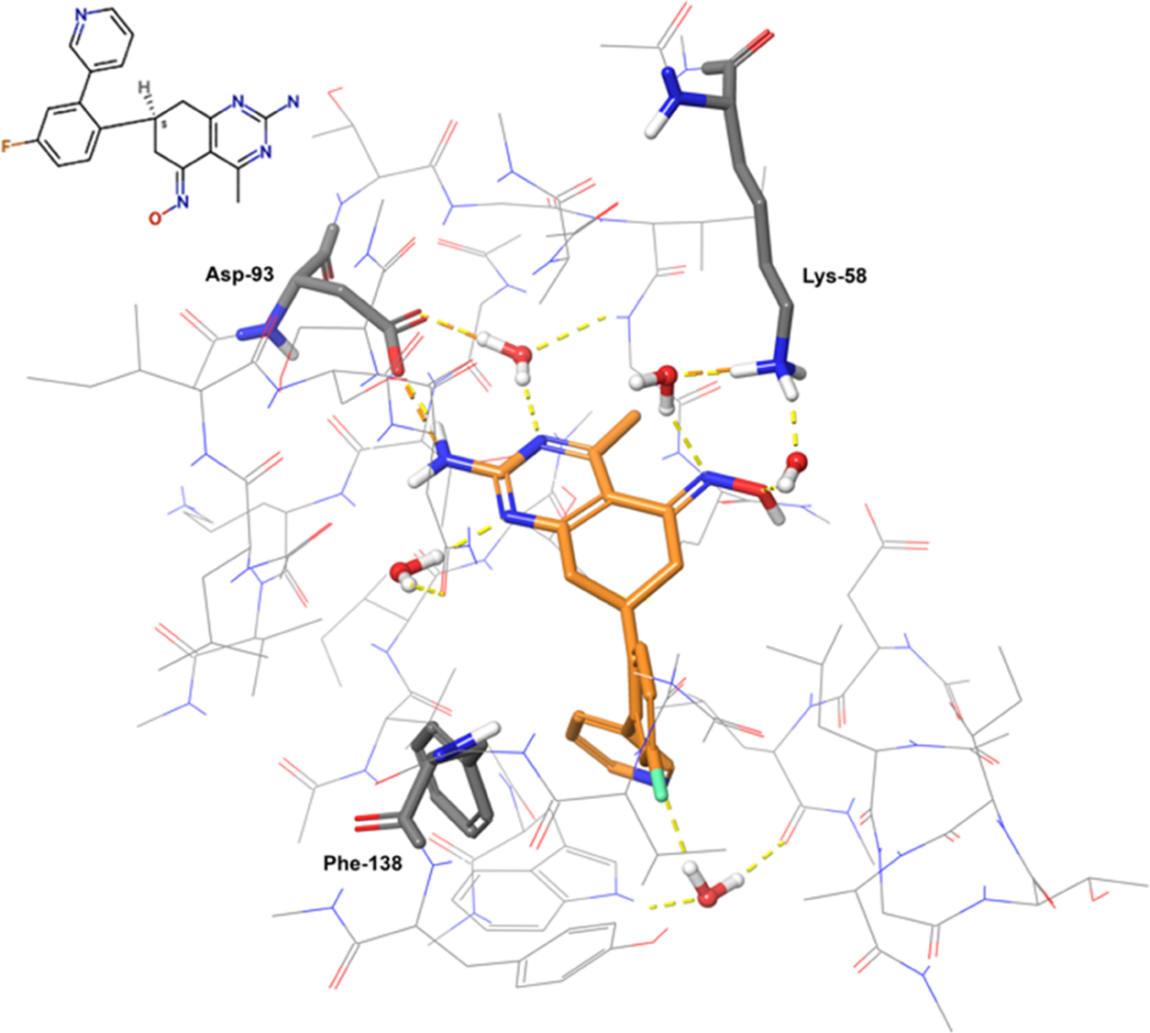
Binding pose of HSP90_lig19 in HSP90 (PDB ID 3FT8). The ligand is represented by orange sticks and the protein by thin gray lines. The protein residues establishing direct contacts with the ligand are shown in gray sticks. Nonpolar hydrogens are not shown. Only the water molecules directly interacting with the ligand are shown.

This series contains a set of 13 ligands that explore scaffold modifications of compound HSP90_lig19, co-crystallized in PDB ID 3FT8 (Figure S5). The substitutions explore biaryl modifications occupying the “helical pocket” in the proximity of the adenosine binding site in HSP90. The experimental binding pose of HSP90_lig19 is stabilized by a vast H-bond network mediated by several water molecules that bridge H-bond donor/acceptor sites on the ligand with the residues of the binding pocket. Other key interactions are established with Lys-58, Asp-93, and Phe-138. As in the case of DNA ligase, all of the water molecules (19 in this case) are retained in the FMO calculations. The binding pose is shown in Figure 7.

**Figure 8:**
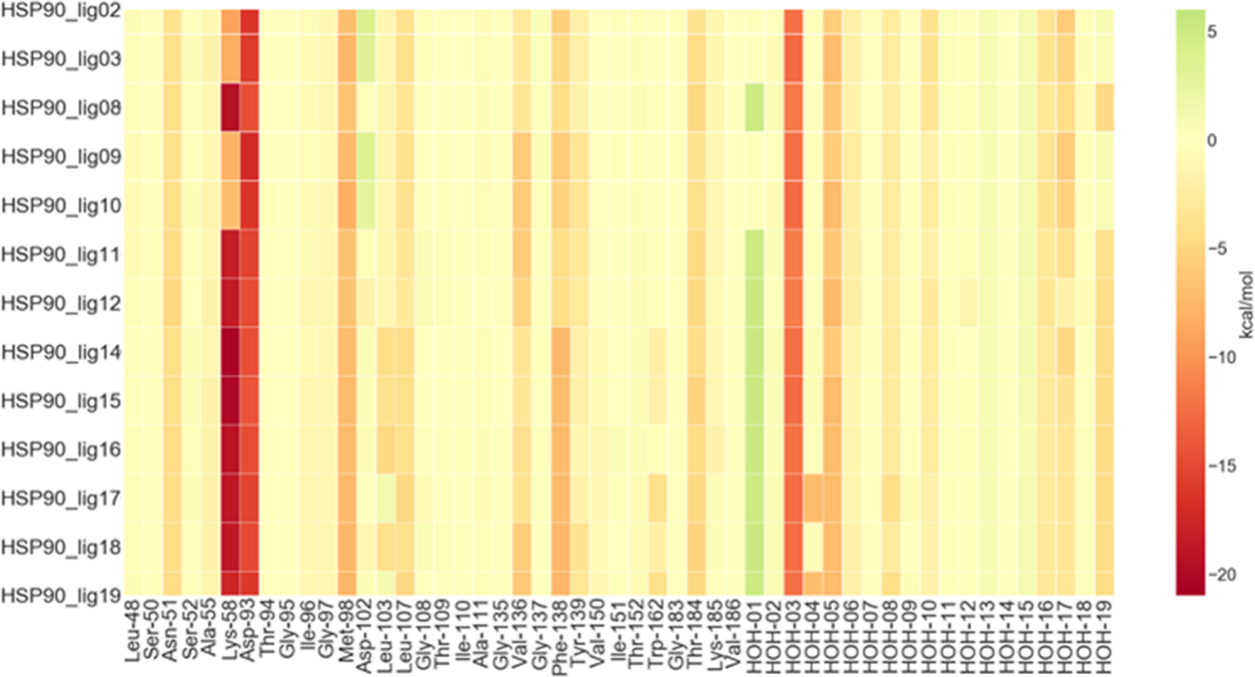
Ligand–protein interaction energy heat map of HSP90. The values plotted (kcal/mol) are obtained from eq 3, where one of the two indexes corresponds to the ligand and the other index to the fragments of the system.

The FMO-based protein–ligand interaction fingerprint map (Figure 8) shows 9 key contacts conserved across all of the ligands (Asn-51, Lys-58, Asp-93, Met-98, Leu-107, Phe-138, Thr-184, HOH3, HOH5). For HSP90_lig02, HSP90_lig03, HSP90_lig09, and HSP90_lig10, the interaction with Lys-58 is weakened by the substitution of the oxime group with a carbonyl. The same substitution leads to a weakly repulsive interaction with Asp-102.

For this system, the variation of affinity across the ligands is directly linked to an enthalpic effect, which is also shown by the strong correlation between the Δ*G*_exp_ and the FMO interaction energy (Figure S6-A). On the other hand, there is a weaker correlation between the experimental activity and clog *P* (Figure S6-B), as is also suggested by the *p*-value of the coefficient in the linear fit, which does not pass the conventional threshold for statistical relevance (*p* > 0.05).

**Figure 9:**
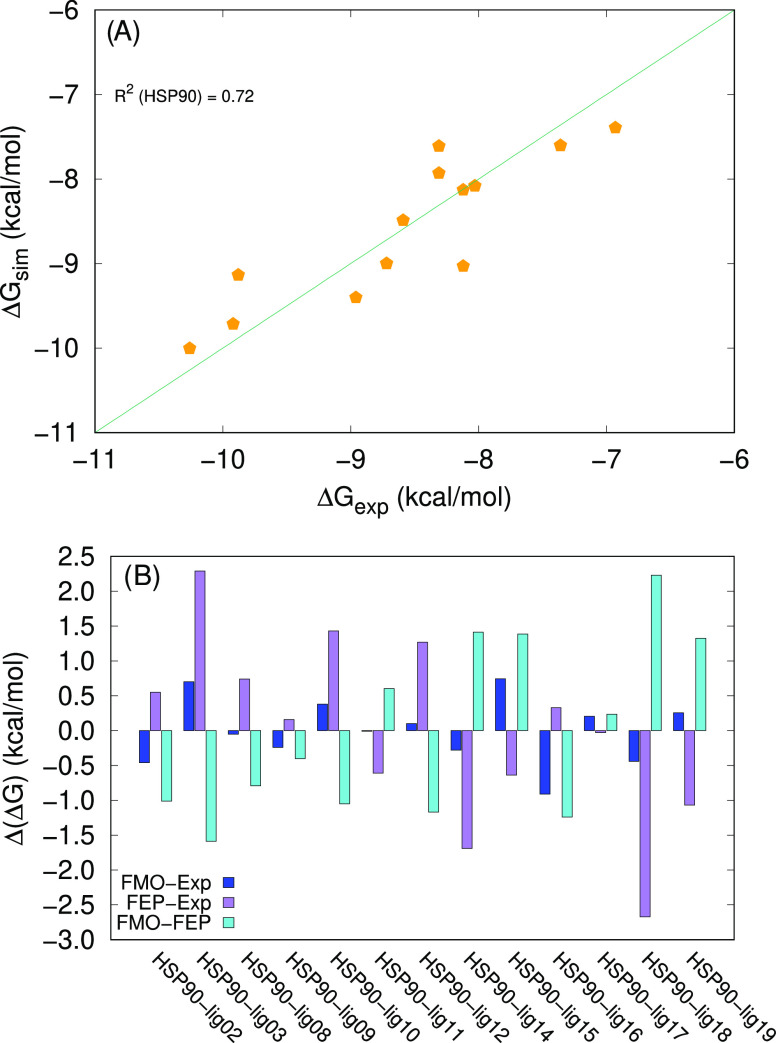
(A) Correlation between FMO-based simulated values and experimental Δ*G* values for HSP90. The diagonal line corresponds to Δ*G*_exp_ =Δ*G*_sim_. (B) Differences between the FMO-based predictions (our data), the FEP-based predictions, and Δ*G*_exp_ for HSP90. The experimental values and the FEP-based predictions are taken from ref (32).

The predictions based on these data are in satisfactory agreement with the experimental values, with *R*^2^ = 0.72 and an average absolute error in the order of 0.4 kcal/mol (Figure 9A and B, respectively). It is interesting to notice that for eight ligands in the selection, namely, HSP90_lig03, HSP90_lig08, HSP90_lig10, HSP90_lig11, HSP90_lig12, HSP90_lig14, HSP90_lig18, and HSP90_lig19, the FMO-based prediction is in closer agreement with the experimental value than the FEP-based results. For HSP90_lig16, instead, the FEP prediction outperforms the FMO-based value. For the other ligands, the two methods produce results of compatible accuracy.

#### p38 α MAP Kinase (p38)

This series contains a set of 6 ligands that explore scaffold modifications of lig01, co-crystallized in PDB ID 1W7H. The complex containing p38_lig05 is obtained instead from PDB ID 1WBW (Figure S7). The substitutions involve the phenyl ring in the structure of p38_lig01 and the positions 2 and 6 in the pyridine ring.

**Figure 10:**
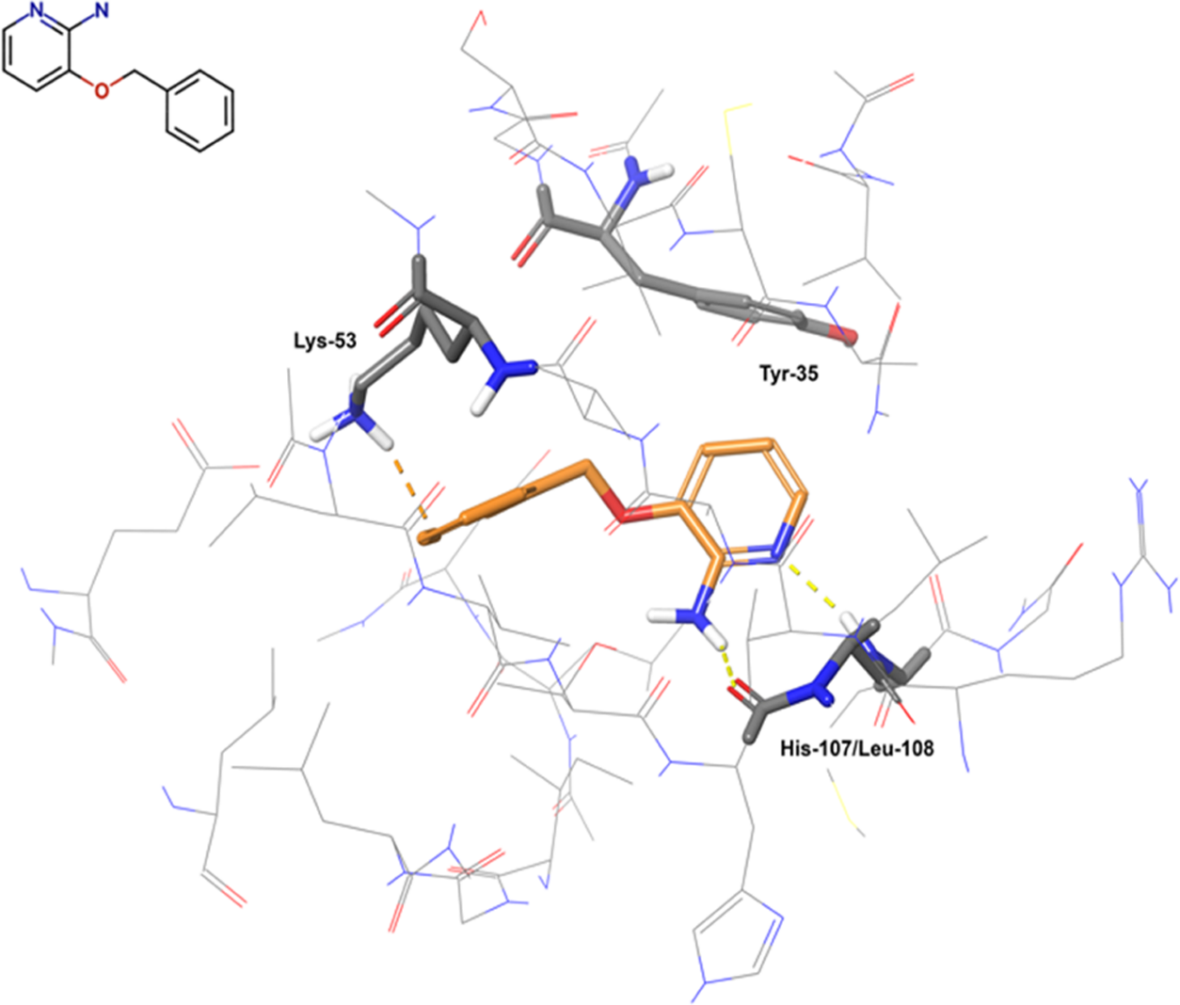
Binding pose of p38_lig01 in p38 (PDB ID 1W7H). The ligand is represented by orange sticks and the protein by thin gray lines. The protein residues establishing direct contacts with the ligand are shown in gray sticks. Nonpolar hydrogens are not shown. Only the water molecules directly interacting with the ligand are shown.

The experimental binding pose of p38_lig01 is stabilized by three key contacts with the protein, namely, a π-stacking with Tyr-35, a π–π–cation interaction with Lys-53, and two H-bonds with the backbone of His-107 and Leu-108. The pose is shown in Figure 10. Notice that, due to the fragmentation scheme in the FMO setup described in the Computational Details section, an FMO fragment does not exactly correspond to an amino acid residue. Each FMO fragment contains the side chain, the backbone NH and C_α_ of one residue plus the carbonyl group of the adjacent residue. Hence, the backbone interactions are shifted by one carbonyl group, and therefore these contacts are assigned to Leu-108 and Met-109 in the interaction heatmap. Three water molecules present in the experimental structure are retained in the FMO calculations, but they are not engaged in key stabilizing interactions with the ligands.

**Figure 11:**
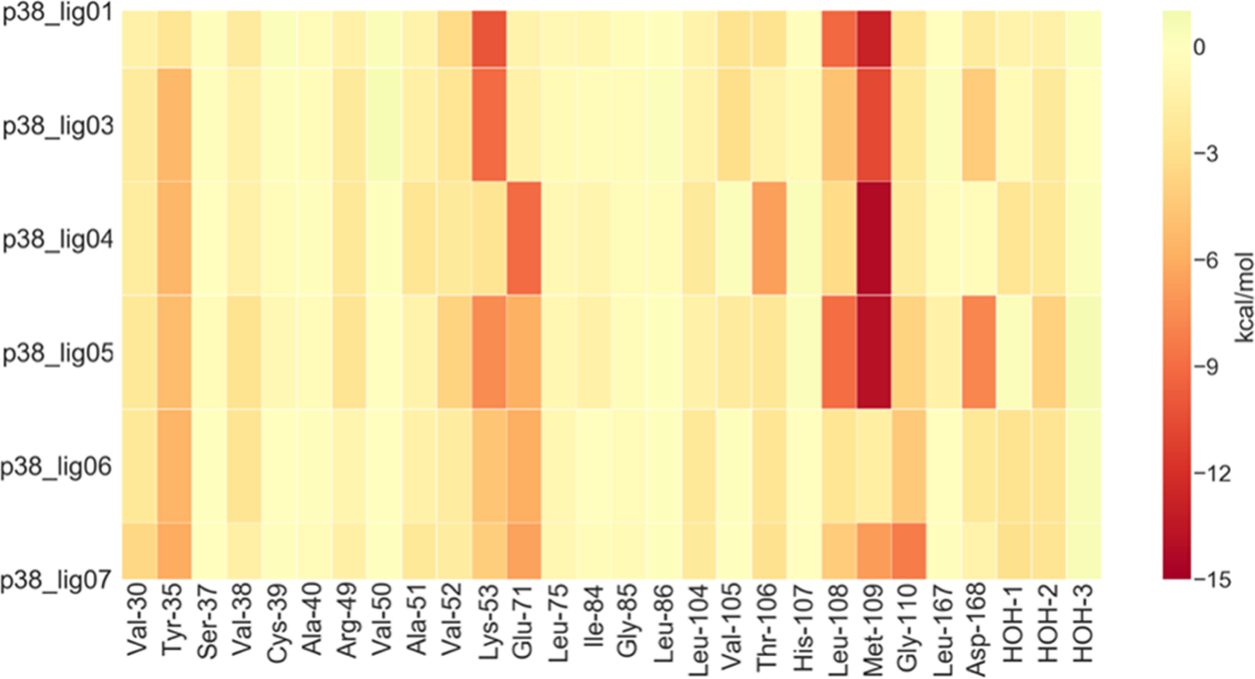
Ligand–protein interaction energy heat map of p38. The values plotted (kcal/mol) are obtained from eq 3, where one of the two indexes corresponds to the ligand and the other index to the fragments of the system.

The interaction fingerprint reveals some inconsistencies across the different ligands considered, as can be observed in Figure 11. There are three main hotspots engaged: Val-30 and Tyr-35, the ionic pair Lys-53/Glu-71, and, as mentioned above, the backbone of Leu-108/Met-109. All ligands engage in the same way the pair Val-30/Tyr-35. p38_lig03 maintains the π–cation interaction with Lys-53 as observed in the X-ray structure of the analogue p38_lig01. The introduction of two chlorine atoms on the phenyl ring induces a positive polarization of the part of the ring pointing toward Glu-71, resulting in a stronger dipole–charge interaction in p38_lig04, p38_lig06, and p38_lig07. Also, in p38_lig05 the naphthalene ring is engaged in strong contact with Glu-71 even if it lacks polarizing substituents on the scaffold. Depending on the replacements in position 6 on the naphthalene, different magnitudes in the interactions with Leu-108/Met-109 are observed.

The correlation between Δ*G*_exp_ and the FMO interaction energy is quite poor, as shown in Figure S8-A. This might be due to a relatively small range of Δ*G*_exp_ which falls in a narrow window of approximately 2.4 kcal/mol. Moreover, for p38_lig06 and p38_lig07 we notice that the substituent introduced on the position 6 of the naphthalene ring points toward a solvent-exposed area and therefore a higher flexibility of the ligand as well as in the protein residues falling in that area could be expected.

On the other hand, there is a stronger correlation with the clog *P* term (Figure S8-B), suggesting that the increase in lipophilicity better represents the increase in experimental affinity.

**Figure 12:**
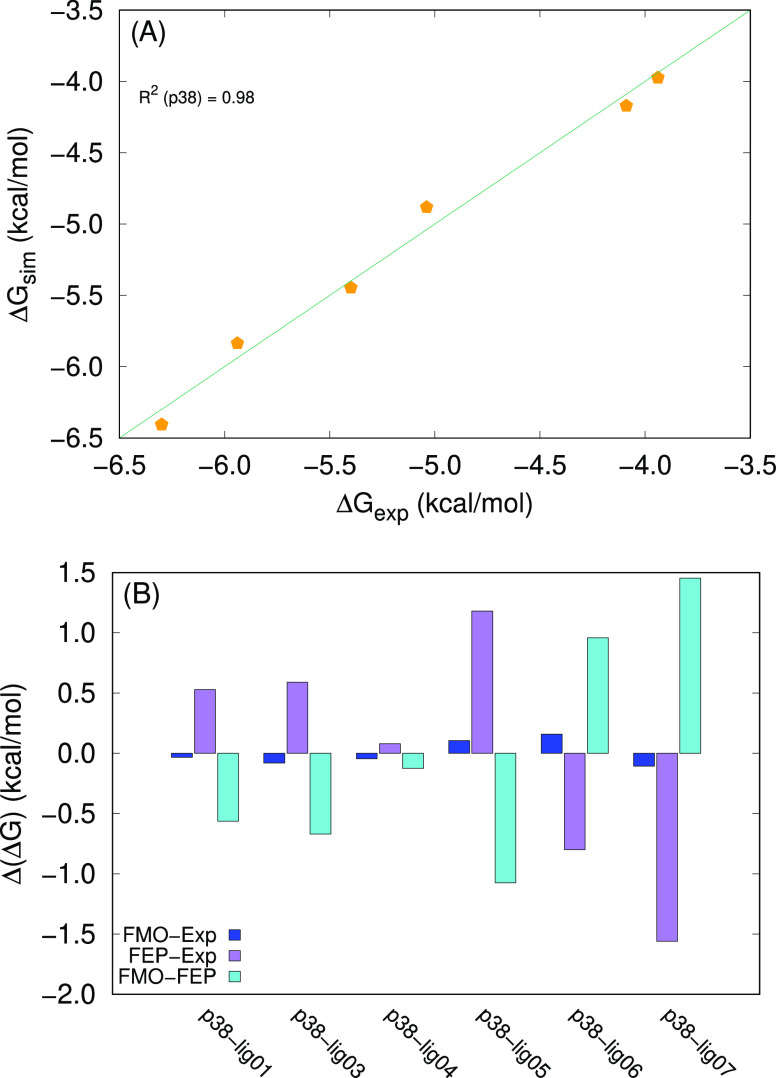
(A) Correlation between FMO-based simulated values and experimental Δ*G* values for p38. The diagonal line corresponds to Δ*G*_exp_ = Δ*G*_sim_. (B) Differences between the FMO-based predictions (our data), the FEP-based predictions, and Δ*G*_exp_ for p38. The experimental values and the FEP-based predictions are taken from ref (32).

If we combine the two terms, the predictions produce an extremely high adjusted *R*^2^ ≈ 0.98, which is a substantial difference with respect to the FEP predictions for which *R*^2^ ≈ 0.67. For all of the ligands, the deviations of the affinity values computed with our approach and the experimental values are lower than 0.1 kcal/mol in absolute value, as shown in Figure 12.

#### JAK-2

**Figure 13:**
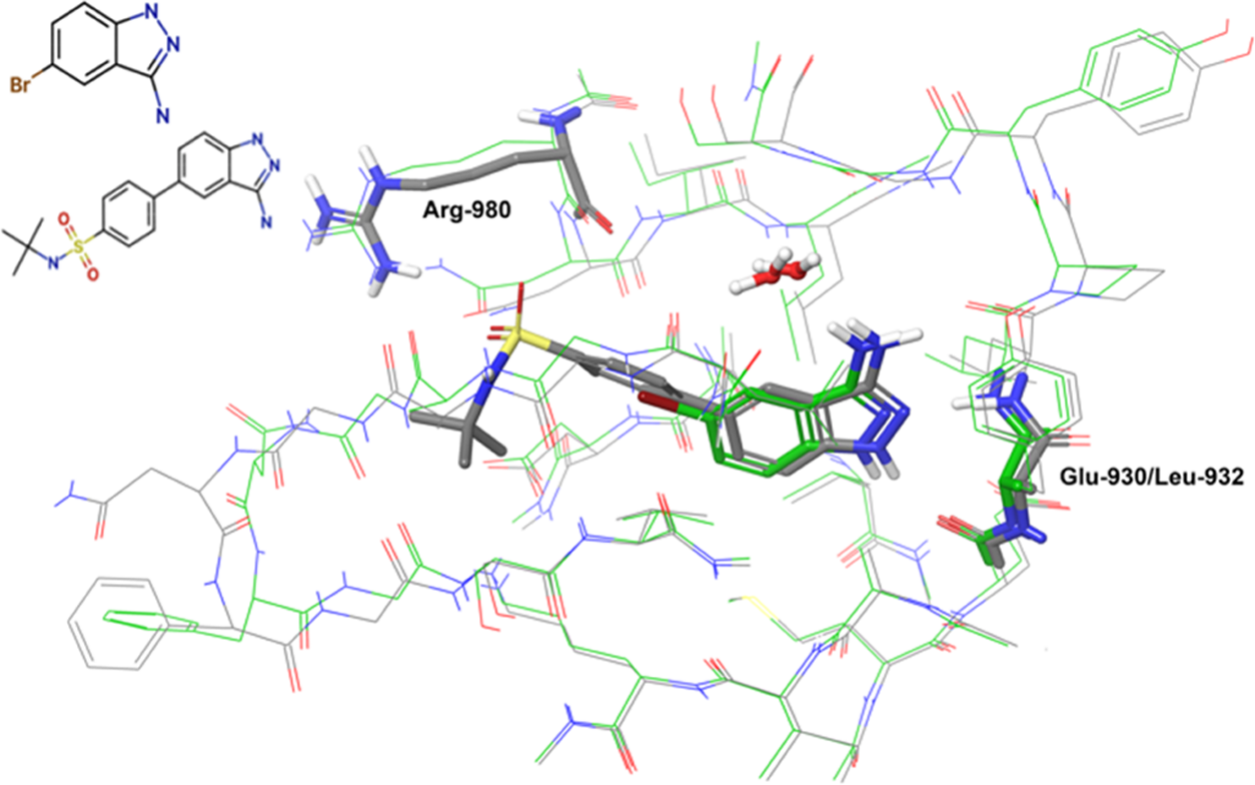
Binding poses of JAK2_lig01 (in green) and JAK2_lig13 (in gray) in two different structures of JAK-2 (PDB ID 3E62, 3E64, respectively). The ligand is represented by sticks and the protein by gray thin lines. The protein residues establishing direct contacts with the ligands are shown in gray sticks. Nonpolar hydrogens are not shown. Only the water molecules directly interacting with the ligand are shown.

This series contains a set of 18 ligands. There are two entries in the protein data bank containing complexes of JAK-2 with ligands, whose scaffold is modified across this series of compounds. PDB ID 3E62 corresponds to a complex with compound jak2_lig01 and PDB ID 3E64 corresponds to a complex with jak2_lig13. The two scaffolds significantly differ, with jak2_lig13 being a more optimized and inhibitor with a larger molecular weight than jak2_lig01. The subset of compounds jak2_lig02 to jak_lig10 explores scaffold modifications of jak2_lig01, where the Br atom is replaced by various heteroaromatic rings. The remaining ligands represent scaffold modifications that start from jak2_lig13 (Figure S9). For this reason, we choose to use two different protein structures for our calculations: PDB ID 3E62, for the subset jak2_lig01to jak_lig10; and PDB ID 3E64, for the subset jak2_lig12 to jak_lig22. Based on this approach and the different sizes of the ligands here considered, we also choose to retain the single water molecule that directly interacts with the ligands in the two experimental structures, which is located in the proximity of the amino group of the 5-amino pyrazole ring. The binding poses of the two experimental structures from which we start our calculations are shown in Figure 13.

**Figure 14:**
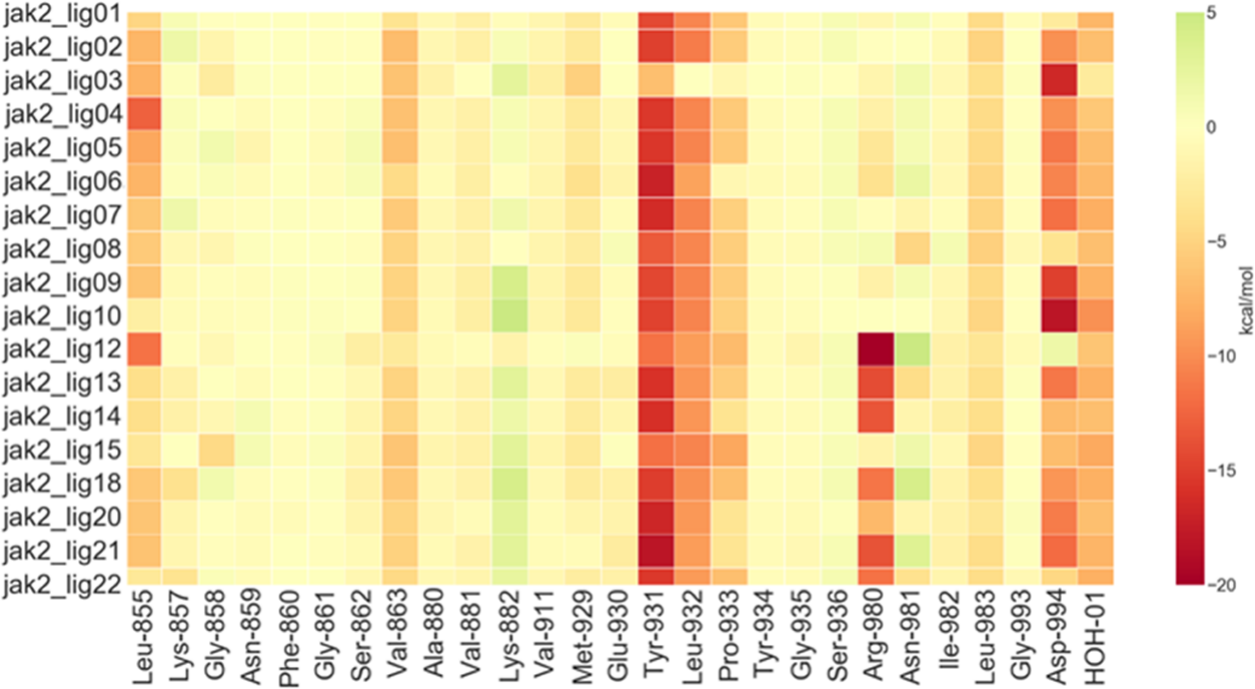
Ligand–protein interaction energy heat map of JAK-2. The values plotted (kcal/mol) are obtained from eq 3, where one of the two indexes corresponds to the ligand and the other index to the fragments of the system.

In the two experimental structures, the aromatic rings, which are deeply embedded in the binding site, occupy the same position and interact in the same way with the backbone of Glu-930 and Leu-932, in addition to the interaction with a conserved water molecule as mentioned above. In our ligand heatmap (Figure 14), due to the FMO fragmentation scheme described in the Computational Details section, the backbone interaction with Glu-930 is assigned to Tyr-931. These interactions are preserved across all of the ligands included in this study. In addition to this, the subset jak2_lig12-22 shows a strong electrostatic attraction between Arg-980 and the sulfonamide groups of the ligands. Among these ligands, this interaction is only absent for jak2_lig15, which indeed does not have a sulfonamide group or other negatively charged atoms in proximity to the side chain of Arg-980.

The correlation of the Δ*G*_exp_ with the clog *P* values of the compounds is very small, while there is a slightly better correlation with the FMO-computed interaction energies, which stays in the order of 0.46 (Figure S10). Combining the two terms, the predictions have adjusted *R*^2^ ≈ 0.59, which is slightly below the results obtained by FEP predictions, which report a correlation of 0.64.

**Figure 15:**
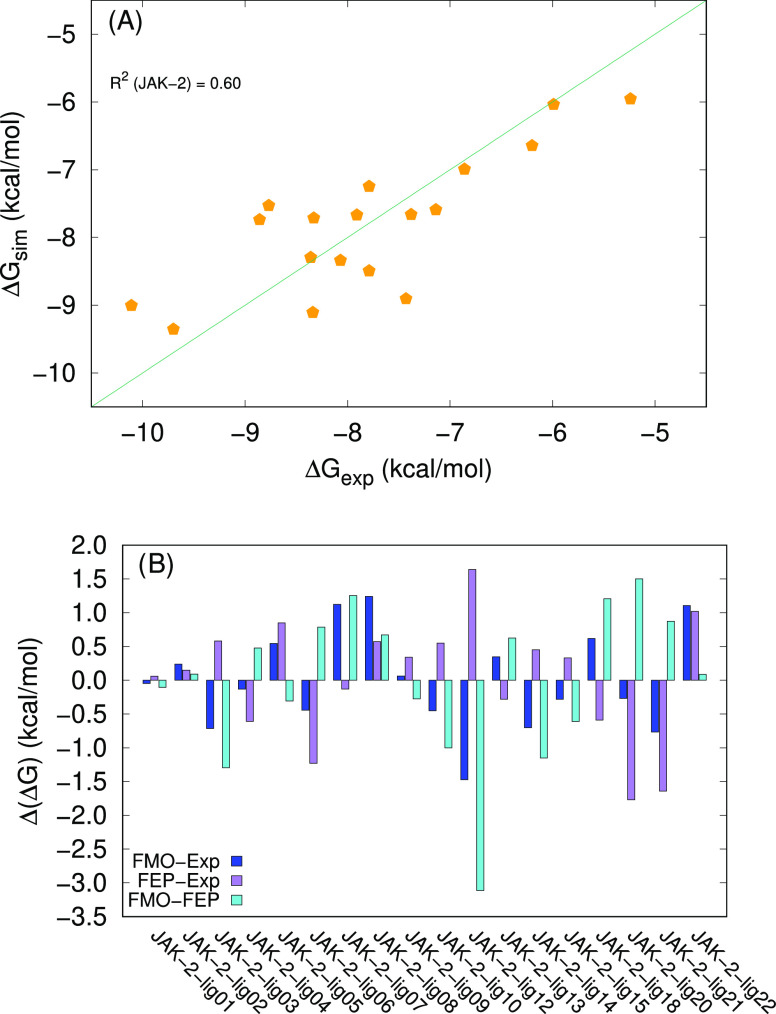
(A) Correlation between FMO-based simulated v alues and experimental Δ*G* values for JAK-2. The diagonal line corresponds to Δ*G*_exp_ = Δ*G*_sim_. (B) Differences between the FMO-based predictions (our data), the FEP-based predictions, and Δ*G*_exp_ for JAK-2. The experimental values and the FEP-based predictions are taken from ref (32).

The predictions for this system turn out to be particularly sensitive to the choice of protein structure. Any attempt at predicting the affinity of all of the 18 compounds using only one of the two structures available as starting point produces extremely weak correlations. For the largest ligands (jak2_lig12 to jak2_lig22), the backbone of the protein opens up to accommodate the full length of the molecules. This side of the protein tends to fold to close the binding pocket when considering smaller ligands (jak2_lig01 to jak2_lig10). This movement is particularly evident if one compares the residues 856-859 between PDB ID 3E62 and 3E64. The flexibility in this part of the protein can be critical for the interaction with the ligands in the subset jak2_lig12-22. Indeed, for this selection of compounds only, the correlation with the experimental values is still unsatisfactory. Only by adding in the results predicted for jak2_lig0110 the correlation is increased (Figure 15).

It is also worth observing that the majority of the inhibitors fall in a narrow activity range of about 2 kcal/mol (between approximately −9 and −7 kcal/mol). As mentioned in the Discussion and Conclusions section, this distribution of the experimental data points complicates the fitting, representing a source of noise.

#### MCL-1

This series contains a set of 15 fragments that explore scaffold modifications of the ligand co-crystallized in PDB ID 4HW3 (Figure S11). This structure corresponds to the complex between MCL-1 and a large ligand bearing the fragment scaffold modified in this series. The substitutions involve different functionalizations of benzothiophene-2-carboxylic acid (mcl_lig01-05), benzofuran-2-carboxylic acid (mcl_lig0611), and benzopyrrole-2-carboxylic acid (mcl_lig12-15). The functional groups added to the different scaffolds involve halogen atoms (Cl and Br) and methyl groups. Therefore, the main difference across the 15 compounds lies essentially in their lipophilicity and in the heteroatom present on the aromatic moiety.

In this system, we followed a slightly different approach to generate the geometries on which the FMO calculations have been performed. The docking grid built on the reference structure does not allow an ideal placement of the aromatic ring of mcl_lig15 (Figure S11), orienting this toward the solvent to avoid a steric clash with Leu-267. Since this geometry does not seem a reasonable choice, we decided to overcome this problem by generating a starting model geometry by hand modifying the co-crystallized ligand. This structure is then minimized without constraints. For internal consistency, all of the geometries of the other complexes are generated in this way. We observe that all of the poses generated by docking are very close to the one produced by manually modifying the ligand present in the experimental structure. This confirms that our binding poses are reasonable and can be confidently used for these calculations.

This dataset contains three ligands (mcl_lig01, mcl_lig06, and mcl_lig12) lacking an exact value of experimental binding affinity. For these compounds only an upper bound energy value of −4.12 kcal/mol has been measured. This value has been used as an experimental data point in the fitting. Moreover, we observe that the affinity range is relatively narrow, spanning about 2 kcal/mol if we take −4.12 kcal/mol as the highest binding energy. This can represent a challenge for our approach for the reasons detailed in the Discussion and Conclusions section.

**Figure 16:**
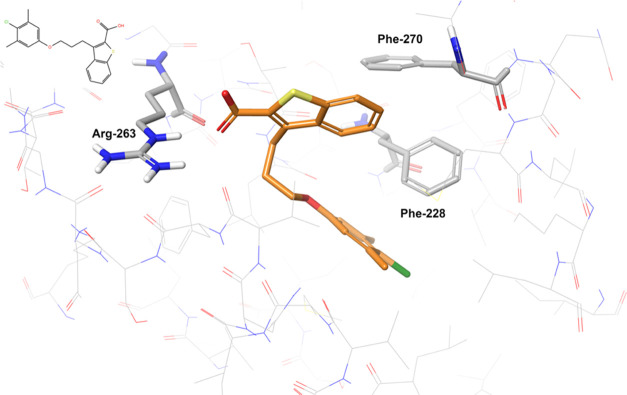
Binding pose of the ligand present in PDB ID 4HW3. This ligand is not included in the dataset studied to generate free energy values. The ligand is represented by orange sticks and the protein by thin gray lines. The protein residues establishing direct contacts with the ligand are shown in gray sticks. Nonpolar hydrogens are not shown.

The experimental structure shown in Figure 16 reveals the key interactions that are observed across the 15 ligands here studied. The dominant contact is clearly the ionic pair between the cationic side chain of Arg-263 and the carboxylic group of the ligand. In addition to this, the aromatic ring is surrounded by Phe-228 and Phe-270 which create a cage of T-shaped π-stacking interaction. The sulfur atom is oriented toward the solvent, leaving the remaining of the molecule deeply embedded in the binding pocket. In this regard, we notice that the fragments considered have much smaller substituents on the scaffold and therefore they could exhibit a certain degree of movement around the position defined by the crystallographic pose.

Apart from the case of mcl_lig15, previously discussed, we notice that mcl_lig13 and mcl_lig14 can be placed in the cavity in two different poses: The first pose overlaps the nitrogen atom of the ligand on the sulfur atom present in the X-ray structure, thus leaving the methyl group exposed to the solvent. The other pose orients the methyl group inside the cavity and can be considered as a rotation of 180 degrees around the longitudinal axis of the molecule of the previously described pose. The latter solution seems slightly more stable than the first pose described.

**Figure 17:**
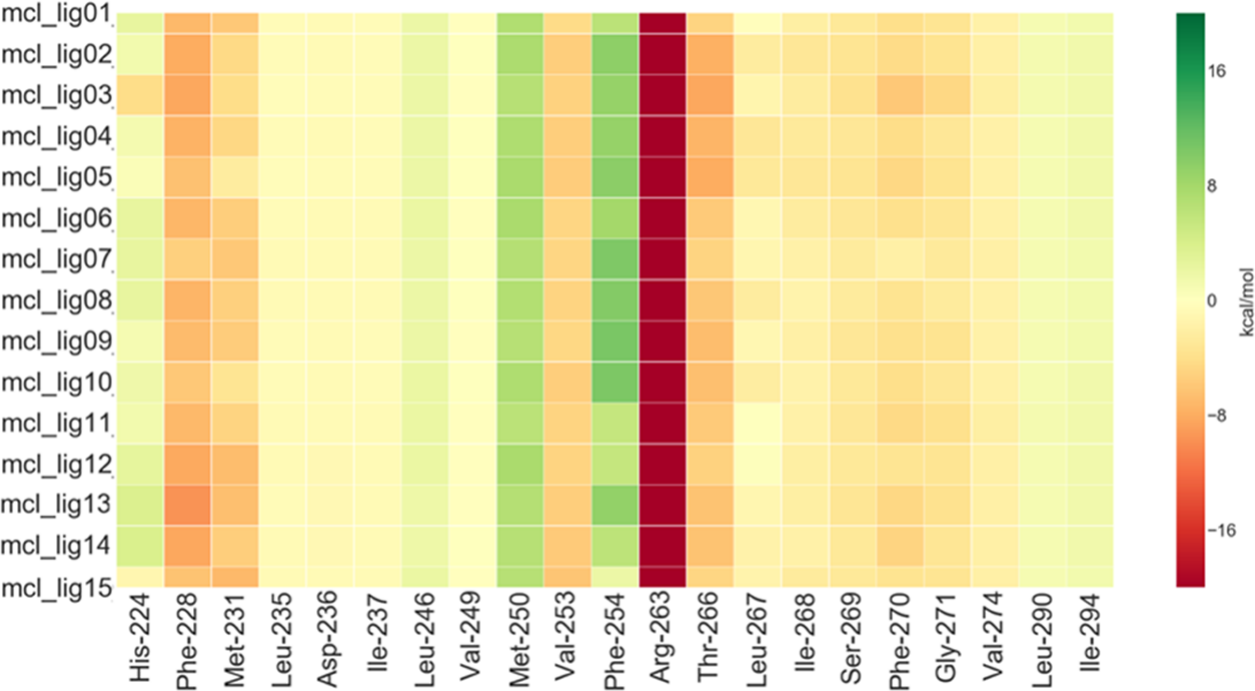
Ligand–protein interaction energy heat map of MCL-1. The values plotted (kcal/mol) are obtained from eq 3, where one of the two indexes corresponds to the ligand and the other index to the fragments of the system.

As expected, the interaction fingerprint heatmap shown in Figure 17 reveals a very conserved pattern, where the contact with Arg-263 is absolutely dominant. In this context, it is worth noting that in the FMO calculations, charge–charge contacts tend to be overestimated in the absence of a solvent. Nonetheless, adding a solvation term using, for instance, the polarizable continuum model (PCM),^5,33,34^ would simply introduce a damping factor in the interaction. Since the fingerprint heatmap is very consistent across all of the ligands considered here, the PCM approach would scale down all of the energies by a similar factor, leaving the general picture unchanged.

The differences across the ligands involve lipophilic moieties and therefore we do not expect a strong correlation between the FMO interaction energy and the experimental binding energy. This is indeed the case, as shown in Figure S12-A. On the other hand, we observe a clearer dependency between experimental binding energy and clog *P*^31^ (Figure S12-B). Our dataset can be divided into three different subsets, depending on the heteroatom present on the ligand core. The experimental affinity is strongly correlated with log *P* in the subset of ligands mcl_lig01/mcl_lig05 (benzothiophene), while it is slightly weaker in the subset mcl_lig05 to mcl_lig11 (benzofuran) and in the subset mcl_lig12 to mcl_lig15 (benzoindole). Overall, our predictions mirror this scenario.

**Figure 18:**
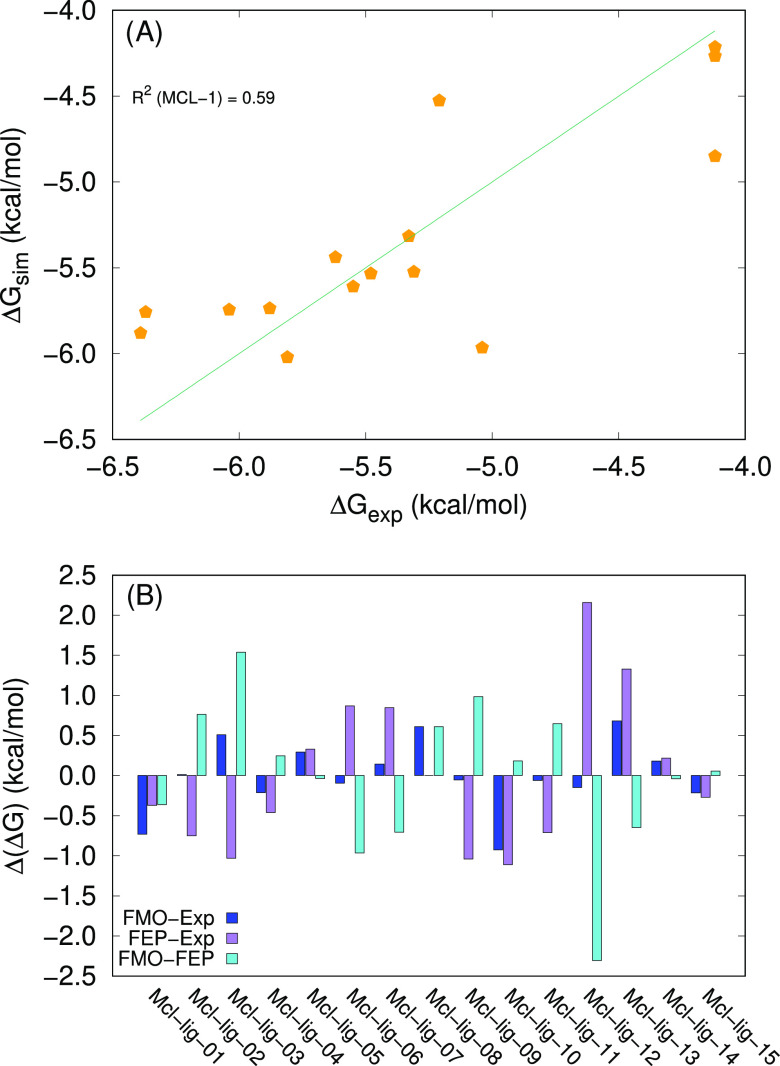
(A) Correlation between FMO-based simulated values and experimental Δ*G* values for MCL-1. The diagonal line corresponds to Δ*G*_exp_ =Δ*G*_sim_. (B) Differences between the FMO-based predictions (our data), the FEP-based predictions, and Δ*G*_exp_ for MCL-1. The experimental values and the FEP-based predictions are taken from ref (32).

The values of Δ*G*_sim_ are dominated by the correlation with clog *P*; the adjusted *R*^2^ is in the order of 0.59 (Figure 18A), with an average absolute error with respect to the experimental values of about 0.32 kcal/mol. The error range is rather wide, encompassing values from 0.01 kcal/mol up to almost 1 kcal/mol (Figure 18B). In this context, we need to recall that three experimental data points are not well defined and that the overall range is quite small. In this situation, the accuracy of the prediction can be strongly reduced

## Discussion and Conclusions

In this work, we have presented SophosQM, a new method to calculate the binding affinity of compounds for a given biological target in a fast, accurate, and reliable way. We validated our approach by predicting the binding energy of a very heterogeneous set of ligands binding to their corresponding targets. In this selection, we encountered different scenarios. The binding could be driven by entropic or enthalpic effects individually or by a combination of the two. Our analysis proved to be efficient at highlighting such binding mechanisms. Overall, our FMO-based binding affinity prediction method shows good performance under two fundamental approximations: the fixed-geometry approach and the use of a macroscopic descriptor, in our case, clog *P*, to incorporate the entropic terms of the binding energy.

**Figure 19:**
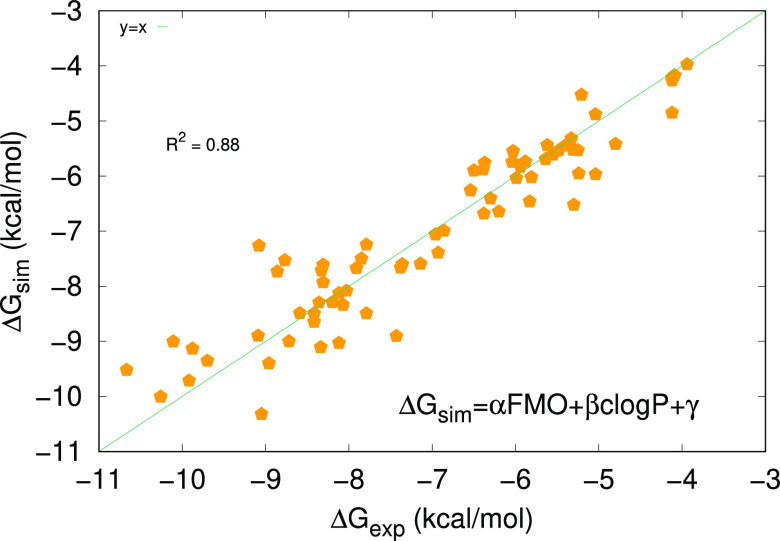
Correlation between Δ*G*_exp_ and Δ*G*_sim_ (FMO) across all of the complexes studied in this work.

Across all of the 70 complexes considered, we observe a very satisfactory correlation with the experimental values, with *R*^2^ ≈ 0.88 (Figure 19). This means that our approach can be successfully employed as an efficient predictive tool. Moreover, in all of the systems, the average error with respect to the experimental value is approximately 0.4 kcal/mol. Therefore, we can say that the method is able to discriminate between active and nonactive compounds. The accuracy is, of course, influenced by the accuracy of the experimental data available. It is interesting to see that the correlation achieved by our approach is comparable to that produced by FEP calculations for the same set of compounds. This should be appreciated as a sign of reliability of our method, showing also that there are experimental datasets and biological targets that are intrinsically more challenging than others. The data presented in this work shows that the QM/FMO method embedded in SophosQM is able to generate reasonably accurate binding affinity predictions. It can also provide an additional layer of information, such as the residue-per-residue heatmaps of interaction energy, and the energy decomposition analysis, which is useful to generate a detailed understanding of the ligand–protein recognition event and guide further molecular design work during the compound optimization process.

Further work on additional and more diverse targets will provide a more robust assessment of the performance of this approach. A systematic evaluation of other QM/semiempirical approximation together with solvent models and molecular descriptors could also lead to an improvement in performance (Table 2).

**Table 2:** List of the Adjusted Correlation Coefficients and the Average Absolute Errors in kcal/mol between Δ*G*_exp_ and Δ*G*_sim_ (FMO) across All of the Systems Studied

system	*R*^2^	⟨|Δ(Δ*G*_sim_^exp^)|⟩ (kcal/mol)
DNA ligase	0.67	0.79
MCL-1	0.59	0.33
MUP-1	0.95	0.16
JAK-2	0.60	0.59
HSP90	0.72	0.37
p38	0.97	0.09

The ability of the method here presented to predict experimental affinity values depends on a set of assumptions that have to be kept in mind while evaluating the results. A key assumption is the geometry of the complexes used for the calculations. In the majority of the cases, the ligands considered represent small modifications of inhibitors that are co-crystallized in the binding pocket. For this reason, we can trust that the binding mode will be preserved, and major conformational rearrangements of the residues in the binding pocket could be safely excluded. However, as discussed in Section E, the possibility of conformational modifications is present when a large ligand branches out toward flexible protein regions. It must be borne in mind that predictions will be more reliable in the context of a congeneric series of compounds sharing the same binding mode.

Another aspect to keep in mind is the resolution of this method in the Δ*G* scale. Since our predictions are ultimately based on a linear fitting, it is important that the experimental data cover a large spectrum, ideally around 5 kcal/mol. If the experimental data encompasses only a small range, the fitting will be biased, and much influenced by the FMO interaction energy values. In this scenario, the FMO method will be less effective in discriminating between additional inhibitors with very similar binding energy and/or very similar interactions with the protein. Obviously, it is also desirable to have an abundance of data points that are equally spread across the whole affinity spectrum so that all of the ranges are equally represented.

Overall, if these requirements are satisfied and if a robust set of fitting parameters is obtained, this approach can be efficiently applied to predict the binding energies of unknown compounds in the design stage. The predictions can be used as a prioritization tool to assist the decision of synthetic chemists, who could direct their efforts toward molecules predicted to have higher binding affinities. This information, combined with appropriate ADME-PK profiling, could in fact save a significant amount of time and resources in a drug discovery compound optimization program. In this light, we want to underline that it is not always necessary to obtain fits with a perfect correlation with the experimental values. For challenging systems, also a moderate correlation would still distinguish good from poor binders.

Finally, the significant increase in speed obtained using FMO-DFTB allows the use of SophosQM beyond compound optimization. It could be used as an accurate scoring function in virtual screening or to analyze molecular dynamics simulations.

## Supporting Information Available

PDB structure, residues forming the binding site, and list of compounds evaluated; correlation plots between compounds Δ*G*_exp_ and FMO interaction energy and clog *P*; numerical values of experimental, FEP-, and FMO-predicted affinity, FMO-derived interaction energy, and calculated log *P*; fitting parameters α, β, and γ; and the RMSD between docked and experimental poses (PDF)
