# Supplementary material for: SophosQM: Accurate Binding Affinity Prediction in Compound Optimization
Source: ACS Omega. 2023 Apr 20;8(17):15083–98. doi: 10.1021/acsomega.2c08132 (PMC10157843; doi:10.1021/acsomega.2c08132)
Supplement: Supplementary file 1 — ao2c08132_si_001.pdf [file ao2c08132_si_001.pdf]

Supporting Information

SophosQM: Accurate Binding Affinity Prediction in Compound Optimization

Riccardo Guareschi, Iva Lukac, Ian H. Gilbert* and Fabio Zuccotto*

Drug Discovery Unit, Wellcome Centre for Anti-Infectives Research, Division of Biological Chemistry and Drug Discovery, University of Dundee, Dow Street, Dundee, DD1 5EH, United Kingdom

Contents

1-6. Protein- Ligand systems

Protein-Ligand systems investigated. For every protein ligand system investigated the following additional information are provided:

A. PDB structure, residues forming the binding site and list of compounds evaluated.
B. Correlation plots between compounds ΔG_{exp} and FMO interaction energy and clogP.
C. Table with numerical values of experimental, FEP-, and FMO-predicted affinity, FMO derived interaction energy and calculated logP.
D. Fitting parameters α, β and γ.

1. DNA Ligase
2. Major urinary protein I (MUP-I)
3. Heat shock protein 90 (HSP90)
4. p38α MAP kinase (p38)

5. Janus Kinase 2 (JAK-2)
6. Myeloid Cell Leukemia 1 (MCL-1)

7. Docking validation

1. DNA Ligase

The complexes are generated from PDB ID 4CC5. This structure corresponds to a complex between DNA Ligase and lig03. The ligand is regarded as neutral and protonated at the position 1 on the triazole ring. Seventeen water molecules are found within the binding site and are retained for the FMO calculations.

The residues included in the FMO calculations are: Met-79, Leu-80, Ser-81, Leu-82, Glu-110, Leu-111, Lys-112, Ile-113, Arg133, Glu-167, Tyr-219, Ser-220, Leu-236, Asp-278, Val-281, Ile-282, Lys-283, Ser-299, Pro-300, Arg-301, Trp-302.

The side chain of Arg-301 is truncated to CH_3 because it points away from the ligand. This selection contains a total of 518 atoms.

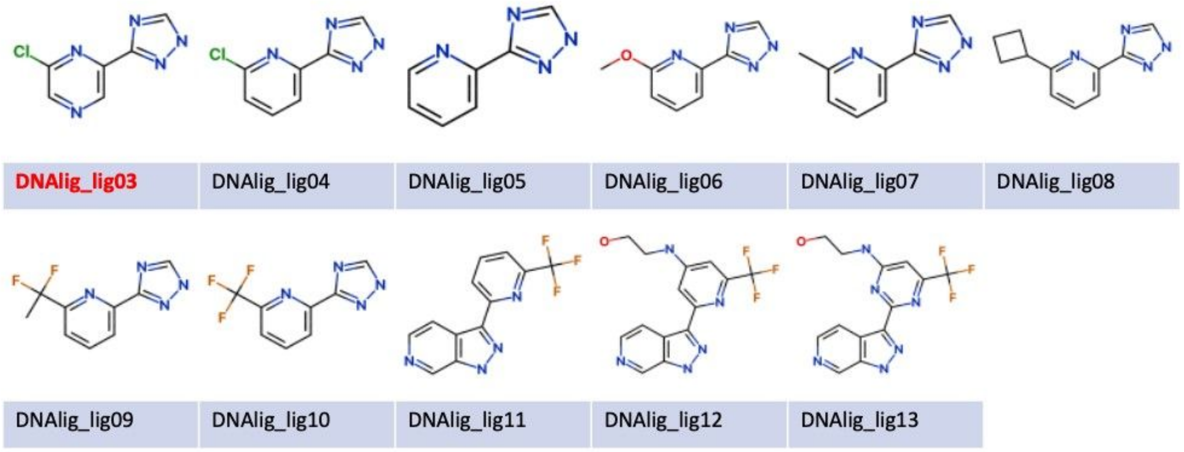

Figure S1: Ligands of DNA ligase included in the study. The ligand highlighted in red corresponds to the one co-crystallised in the experimental structure.

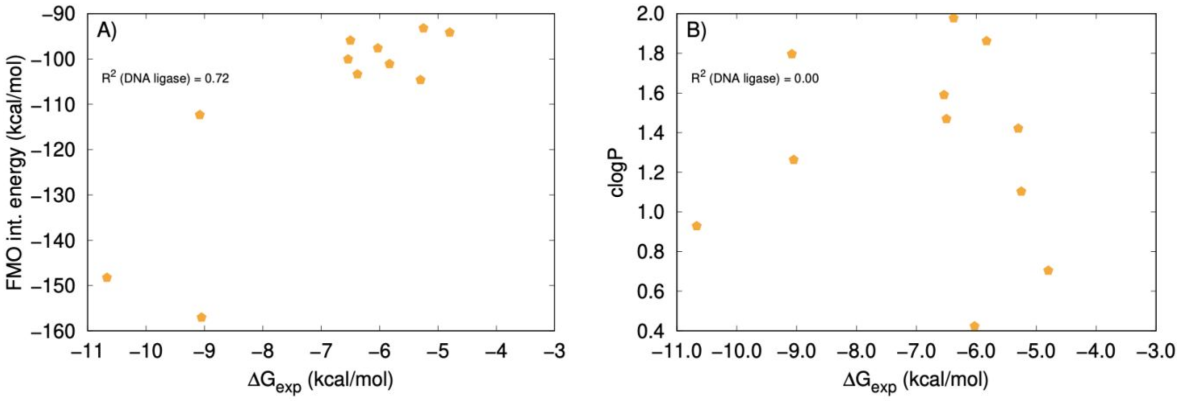

Figure S2: Correlation between ΔG_{exp} and FMO interaction energy (A) and clogP (B) in DNA ligase.

Table S1: Comparison between the experimental, FEP-, and FMO-predicted affinity values in DNA ligase. All energies are expressed in kcal/mol. The fitting parameters to obtain the FMO predictions are: $\alpha = 0.074$, $\beta = -0.456$, $\gamma = 1.856$.

Ligand	ΔG_{exp}	ΔG_{sim}(FEP)	ΔG_{sim}(FMO)	FMO int. energy	clogP
lig03	-6.03	-5.47	-5.55	-97.63	0.42
lig04	-6.5	-5.84	-5.90	-95.91	1.47
lig05	-4.8	-4.06	-5.42	-94.15	0.70
lig06	-5.3	-5.25	-6.52	-104.63	1.42
lig07	-5.25	-4.29	-5.53	-93.22	1.10
lig08	-5.83	-5.4	-6.46	-101.10	1.86
lig09	-6.38	-5.61	-6.68	-103.38	1.98
lig10	-6.54	-6.12	-6.26	-100.07	1.59
lig11	-9.08	-9.26	-7.26	-112.35	1.80
lig12	-9.05	-10.43	-10.32	-157.06	1.26
lig13	-10.67	-13.63	-9.52	-148.28	0.93

2. Major urinary protein I (MUP-I)

The complexes are generated starting from PDB ID 1T06. This structure corresponds to the complex between MUP-I and lig01. Two water molecules are found within the binding site and are retained for the FMO calculations. The residues included in the FMO calculations are: Leu-42, Phe-56, Leu-58, Phe-59, Leu-60, Glu-61, Gln-62, Ile-63, Leu-72, Lys-73, Phe-74, Met-87, Val-100, Thr-101, Tyr-102, Asn-106, Thr-107, Phe-108, Thr-109, Ile-110, Leu-119, Met-120, Ala-121, His-122, Leu-123, Leu-134, Met-135, Gly-136, Leu-137, Tyr-138.

The side chains of Arg-57, Glu-61, and Lys-73 are truncated to CH_3 as they point away from the binding site. This selection contains a total of 618 atoms.

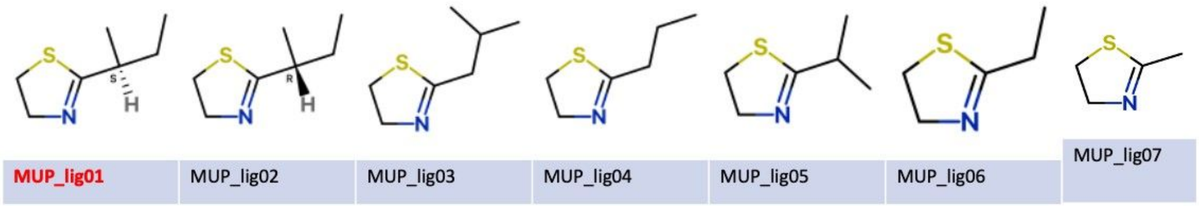

Figure S3: Ligands of MUP-I included in the study. The ligand highlighted in red corresponds to the one co-crystallised in the experimental structure.

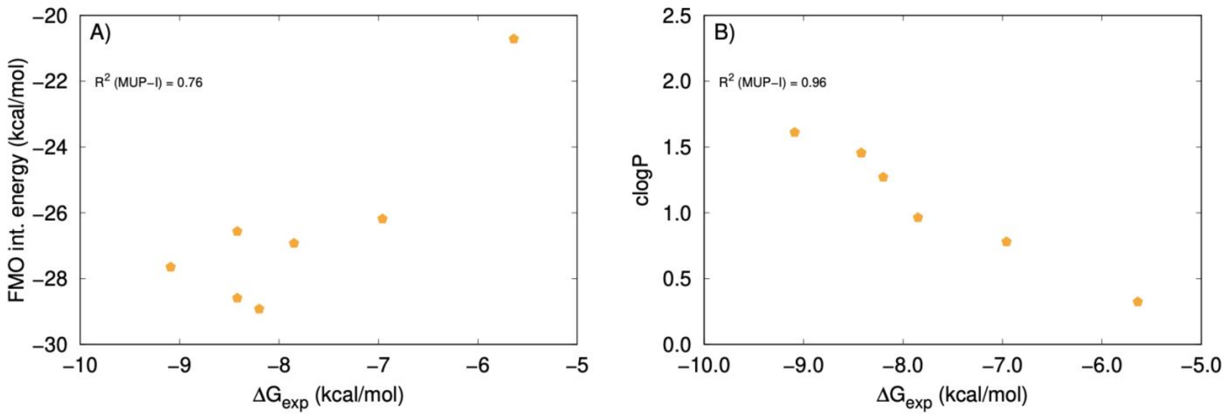

Figure S4: Correlation between ΔG_{exp} and FMO interaction energy (A) and clogP (B) for the MUP-I case. In plot B, only six points appear, since the dataset contains two enantiomers with the same ΔG_{exp}.

Table S2: Comparison between the experimental, FEP-, and FMO-predicted affinity values in MUP-I. All energies are expressed in kcal/mol. The fitting parameters to obtain the FMO predictions are: $\alpha = 0.076$, $\beta = -2.079$, $\gamma = -3.437$.

Ligand	ΔG_{exp}	ΔG_{sim}(FEP)	ΔG_{sim}(FMO)	FMO int. energy	clogP
lig01	-8.42	-8.82	-8.65	-28.59	1.46
lig02	-8.42	-8.82	-8.49	-26.56	1.46
lig03	-9.09	-8.75	-8.90	-27.65	1.61
lig04	-8.20	-7.97	-8.29	-28.93	1.27
lig05	-7.85	-7.74	-7.50	-26.92	0.96
lig06	-6.96	-6.85	-7.06	-26.18	0.78
lig07	-5.64	-5.63	-5.69	-20.72	0.32

3. Heat shock protein 90 (HSP90)

The complexes are generated starting from PDB ID 3FT8. This structure corresponds to the complex between HSP90 and lig19. Nineteen water molecules are found within the binding pocket and are retained for the FMO calculations.

The residues included in the FMO calculations are: Leu-48, Ser-50, Asn-51, Ser-52, Ala-55, Lys-58, Asp-93, Thr-94, Gly-95, Ile-96, Gly-97, Met-98, Asp-102, Leu-103, Leu-107, Gly-108, Thr-109, Ile-110, Ala-111, Gly-135, Val-136, Gly-137, Phe-138, Tyr-139, Val-150, Ile-151, Thr-152, Trp-162, Gly-183, Thr-184, Lys-185, Val-186. This selection contains a total of 662 atoms.

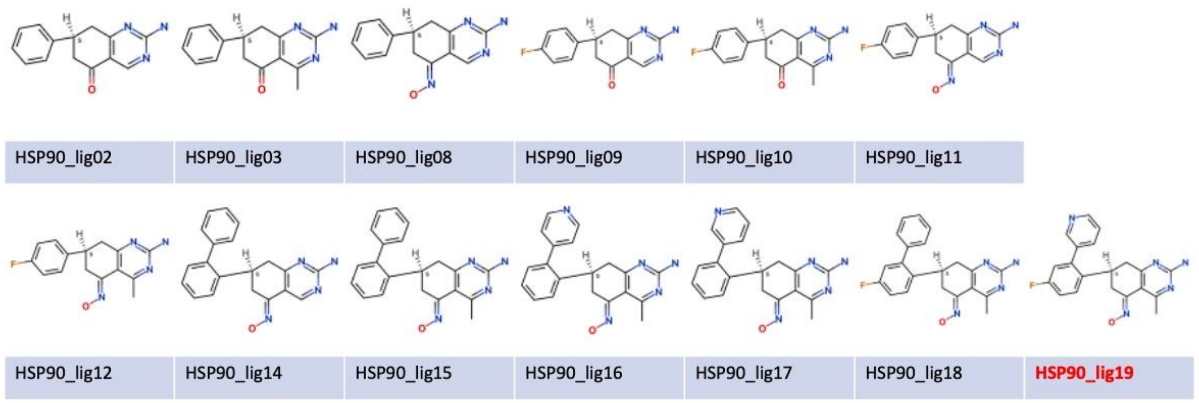

Figure S5: Ligands of HSP90 included in the study. The ligand highlighted in red corresponds to the one co-crystallised in the experimental structure.

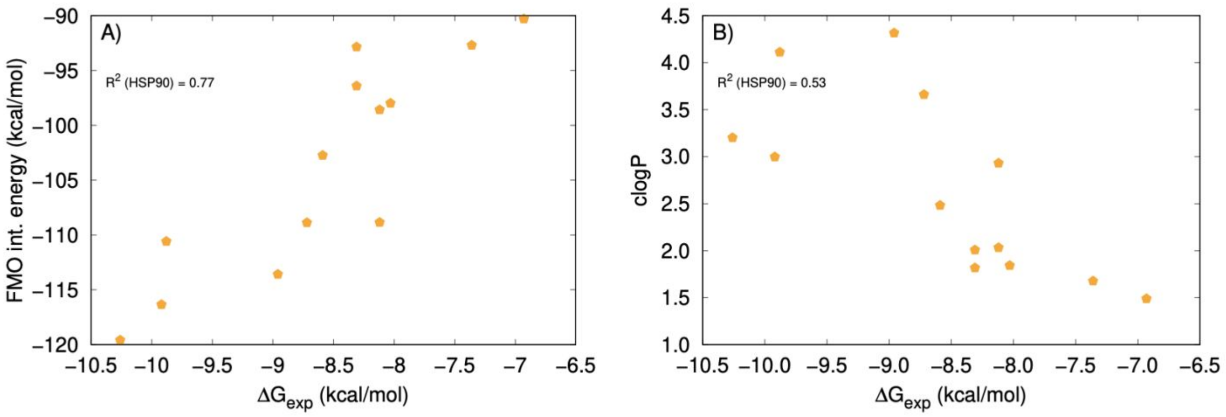

Figure S6: Correlation between ΔG_{sim} and FMO interaction energy (A) and clogP (B) in HSP90.

Table S3: Comparison between the experimental, FEP-, and FMO-predicted affinity values in HSP90. All energies are expressed in kcal/mol. The fitting parameters to obtain the FMO predictions are: $\alpha = 0.092$, $\beta = 0.045$, $\gamma = 0.837$.

Ligand	ΔG_{exp}	ΔG_{sim}(FEP)	ΔG_{sim}(FMO)	FMO int. energy	clogP
lig02	-6.93	-6.38	-7.39	-90.30	1.49
lig03	-8.31	-6.02	-7.61	-92.86	1.82
lig08	-8.03	-7.29	-8.08	-97.99	1.84
lig08	-7.36	-7.20	-7.60	-92.70	1.68
lig10	-8.31	-6.88	-7.93	-96.41	2.01
lig11	-8.12	-8.73	-8.13	-98.58	2.03
lig12	-8.59	-7.32	-8.49	-102.74	2.48
lig14	-8.72	-10.41	-9.00	-108.87	3.66
lig15	-9.88	-10.52	-9.13	-110.57	4.11
lig16	-8.12	-7.79	-9.03	-108.84	2.93
lig17	-9.92	-9.95	-9.72	-116.35	3.00
lig18	-8.96	-11.63	-9.40	-113.57	4.32
lig19	-10.26	-11.33	-10.00	-119.59	3.20

4. p38α MAP kinase (p38)

The complexes are generated starting from PDB ID 1W7H. This structure corresponds to the complex between p38 and lig01. Three water molecules are found within the binding pocket and are retained for the FMO calculations.

The residues included in the FMO calculations are: Val-30, Tyr-35, Ser-37, Val-38 Cys39, Ala-40, Arg-49, Val-50, Ala-51, Val-52, Lys-53, Glu-71, Leu-75, Ile-84, Gly-85, Leu-86, Leu-104, Val-105, Thr-106, His-107, Leu-108, Met-109, Gly-110, Leu-167, Asp-168. This selection contains a total of 504 atoms.

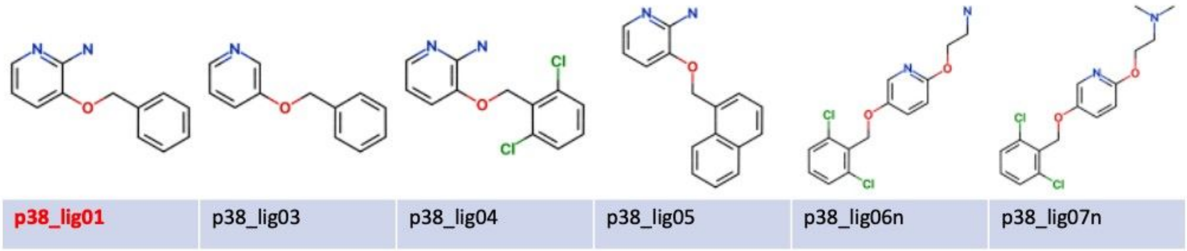

Figure S7: Ligands of p38α MAP kinase included in the study. The ligand highlighted in red corresponds to the one co-crystallised in the experimental structure.

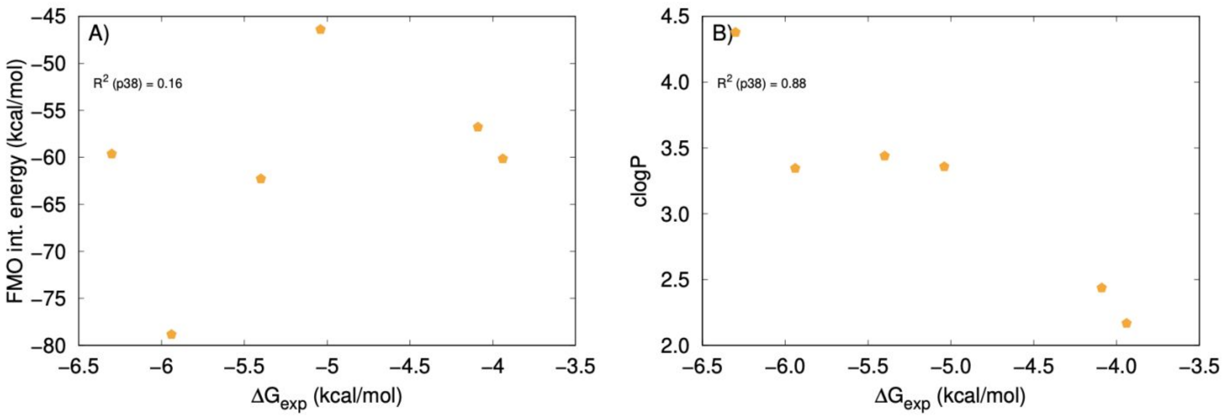

Figure S8: Correlation between ΔG_{exp} and FMO interaction energy (A) and clogP (B) in p38.

Table S4: Comparison between the experimental, FEP-, and FMO-predicted affinity values in p38. All energies are expressed in kcal/mol. The fitting parameters to obtain the FMO predictions are: $\alpha = 0.030$, $\beta = -1.107$, $\gamma = 0.223$.

Ligand	ΔG_{exp}	ΔG_{sim}(FEP)	ΔG_{sim}(FMO)	FMO int. energy	clogP
lig01	-3.94	-3.41	-3.97	-60.15	2.17
lig03	-4.09	-3.50	-4.17	-56.78	2.44
lig04	-5.40	-5.32	-5.44	-62.28	3.44
lig05	-5.94	-4.76	-5.84	-78.84	3.35
lig06	-5.04	-5.84	-4.88	-46.41	3.36
lig07	-6.30	-7.86	-6.41	-59.63	4.38

5. Janus Kinase 2 (JAK-2)

The complexes are generated starting from PDB ID 3E62 and 3E64. The structures correspond to the complexes between JAK-2 and lig01 and lig13, respectively. The subset lig01-lig10 is modeled starting from the 3E62 structure, while the remaining complexes are modeled on the 3E64 structure. Due to the different shapes of the ligands, only one water is kept in the FMO calculations. More details about this choice are discussed in the main text. For lig03, only one run is performed, choosing the geometry that overlaps the indazole ring and the NH_2 group on the co-crystallised ligand. For ligands 4, 6, 7, 8, 9, 10 two different poses are considered. In each case, the two poses differ for a rotation of 180 degrees around the σ-bond connecting the indazole ring and the substituent in position 5. The pose producing the lowest value of FMO interaction is used to build the predictive model.

The residues included in the FMO calculations are: Leu-855, Lys-857, Gly-858, Asn-859, Phe-860, Gly-861, Ser-862, Val-863, Ala-880, Val-881, Lys-882, Val-911, Met-929, Glu-930, Tyr-931, Leu-932, Pro-933, Tyr-934, Gly-935, Ser-936, Arg-980, Asn-981, Ile-982, Leu-983, Gly-993, Asp-994. This selection contains a total of 470 atoms.

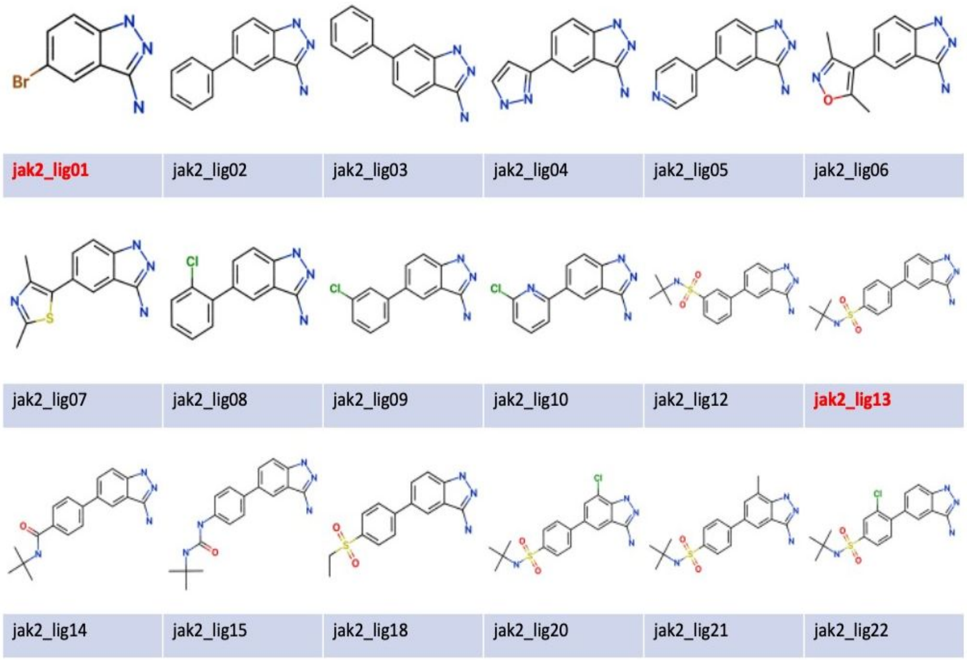

Figure S9: Ligands of JAK-2 included in the study. The ligand highlighted in red corresponds to the one co-crystallised in the experimental structure.

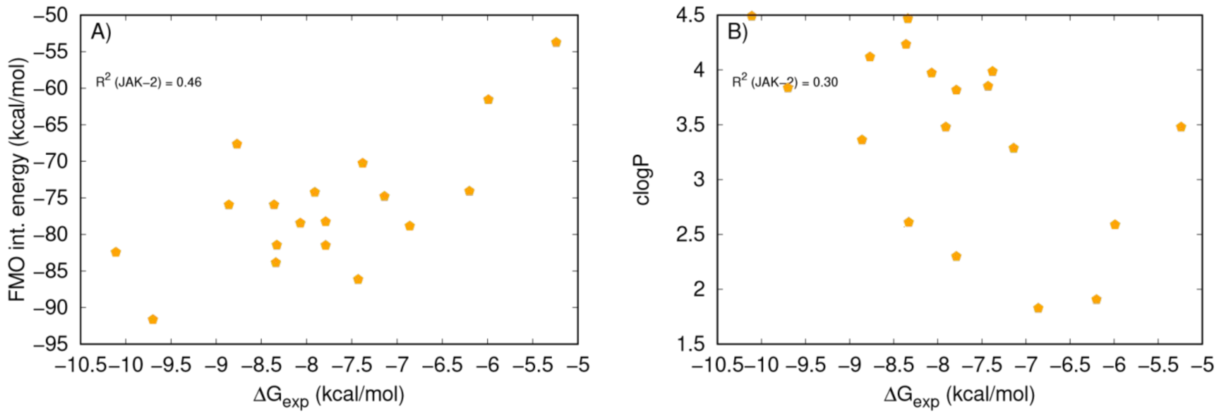

Figure S10: Correlation between ΔG_{exp} and FMO interaction energy (A) and clogP (B) in JAK-2.

Table S5: Comparison between the experimental, FEP-, and FMO-predicted affinity values in JAK-2. All energies are expressed in kcal/mol. The fitting parameters to obtain the FMO predictions are: $\alpha = 0.0840$, $\beta = -0.643$, $\gamma = 0.773$.

Ligand	ΔG_{exp}	ΔG_{sim}(FEP)	ΔG_{sim}(FMO)	FMO int. energy	clogP
lig01	-5.99	-5.93	-6.04	-61.57	2.59
lig02	-7.91	-7.76	-7.67	-74.23	3.48
lig03	-5.24	-4.66	-5.95	-53.72	3.48
lig04	-6.86	-7.47	-6.99	-78.84	1.83
lig05	-7.79	-6.94	-7.25	-78.26	2.30
lig06	-6.20	-7.43	-6.64	-74.07	1.91
lig07	-8.86	-8.99	-7.74	-75.95	3.36
lig08	-8.77	-8.20	-7.53	-67.64	4.12
lig09	-8.36	-8.02	-8.30	-75.94	4.23
lig10	-7.14	-6.59	-7.59	-74.79	3.29
lig12	-7.43	-5.79	-8.90	-86.14	3.85
lig13	-9.70	-9.98	-9.35	-91.64	3.84
lig14	-7.79	-7.34	-8.49	-81.50	3.82
lig15	-7.38	-7.05	-7.66	-70.26	3.99

lig18	-8.33	-8.92	-7.71	-81.45	2.61
lig20	-8.07	-9.84	-8.34	-78.44	3.97
lig21	-8.34	-9.98	-9.11	-83.85	4.47
lig22	-10.11	-9.09	-9.00	-82.43	4.49

6. Myeloid Cell Leukemia 1 (MCL-1)

The complexes are generated starting from PDB ID 4HW3. This structure does not correspond to a complex with any of the ligands considered in the work. No water molecule is included in the binding pocket. The residues included in the FMO calculations are: His-224, Phe-228, Met-231, Leu-235, Asp-236, Ile-237, Leu-246, Val-249, Met-250, Val-253, Phe-254, Arg-263, Thr-266, Leu-267, Ile-268, Ser-269, Phe-270, Gly-271, Val-274, Leu-290, Ile-294.

The side chain of Asp-236 is truncated to CH_3 because it points away from the ligand. Moreover, this avoids charge imbalance in the FMO dimer calculations with the ligand that is anionic. This selection contains a total of 512 atoms.

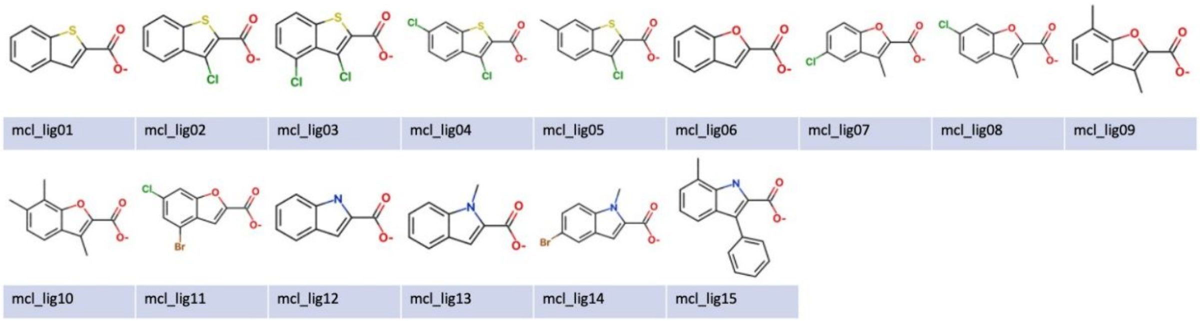

Figure S11: Ligands of MCL-1 included in the study.

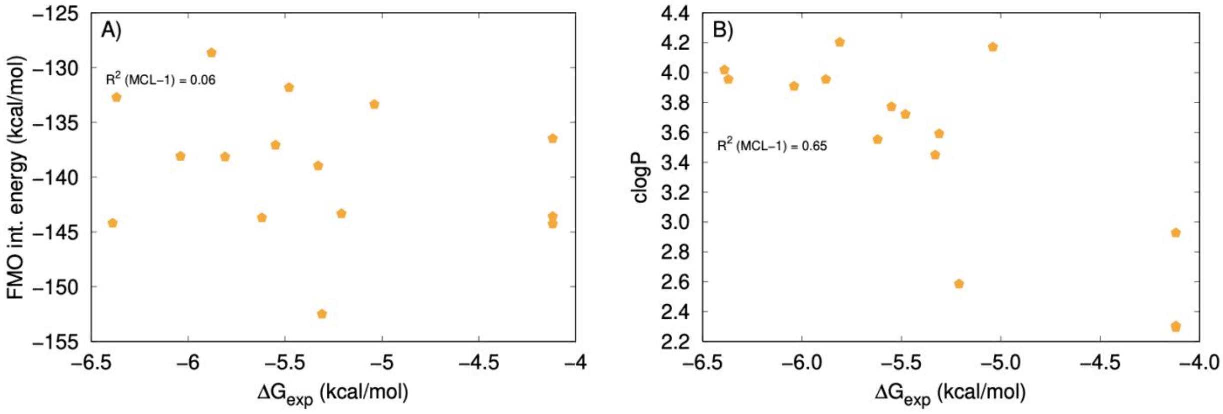

Figure S12: Correlation between ΔG_{exp} and FMO interaction energy (A) and clogP (B) in MCL-1.

Table S6: Comparison between the experimental, FEP-, and FMO-predicted affinity values in MCL-1. All energies are expressed in kcal/mol. The fitting parameters to obtain the FMO predictions are: $\alpha = 0.0054$, $\beta = -0.9418$, $\gamma = -1.31116$.

Ligand	ΔG_{exp}	ΔG_{sim}(FEP)	ΔG_{sim}(FMO)	FMO int. energy	clogP
lig01	-4.12	-4.49	-4.85	-143.58	2.93
lig02	-5.33	-6.08	-5.32	-138.96	3.45
lig03	-6.39	-7.42	-5.88	-144.19	4.02
lig04	-5.81	-6.27	-6.02	-138.15	4.20
lig05	-6.04	-5.71	-5.74	-138.09	3.91
lig06	-4.12	-3.25	-4.21	-136.47	2.29
lig07	-5.88	-5.03	-5.74	-128.65	3.96
lig08	-6.37	-6.37	-5.76	-132.72	3.96
lig09	-5.48	-6.52	-5.53	-131.82	3.72
lig10	-5.04	-6.15	-5.97	-133.36	4.17
lig11	-5.55	-6.26	-5.61	-137.07	3.77
lig12	-4.12	-1.96	-4.27	-144.26	2.30
lig13	-5.21	-3.88	-4.53	-143.33	2.59
lig14	-5.62	-5.40	-5.44	-143.70	3.55
lig15	-5.31	-5.58	-5.52	-152.50	3.59

7 Docking validation

Table S7: RMSD between the ligand co-crystallized in the X-ray structure used as docking grid for each system and the same ligand in the geometry used in the FMO calculation, which is obtained after docking and structural minimization. As mentioned in the main text, the MCL-1 protein structure does not contain a ligand form the evaluated chemical series and therefore is not included in this table.

**System**	**RMSD (Å)**
DNA ligase	0.54
MUP-1	0.33
HSP90	<0.01
p38	0.58
Jak-2	0.37 (PDB ID 3E64)<br>0.12 (PDB ID 3E62)
